# iPLA2β-mediated lipid detoxification controls p53-driven ferroptosis independent of GPX4

**DOI:** 10.1038/s41467-021-23902-6

**Published:** 2021-06-15

**Authors:** Delin Chen, Bo Chu, Xin Yang, Zhaoqi Liu, Ying Jin, Ning Kon, Raul Rabadan, Xuejun Jiang, Brent R. Stockwell, Wei Gu

**Affiliations:** 1 Institute for Cancer Genetics, and Department of Pathology and Cell Biology, and Herbert Irving Comprehensive Cancer Center,Vagelos College of Physicians & Surgeons, Columbia University, New York, NY 10032 USA; 2 Program for Mathematical Genomics, Departments of Systems Biology and Biomedical Informatics, Columbia University, New York, NY 10032 USA; 3 Cell Biology Program, Memorial Sloan-Kettering Cancer Center, New York, NY 10065 USA; 4 Department of Biological Sciences, Department of Chemistry, Columbia University, New York, NY 10027 USA

**Keywords:** Cancer metabolism, Cell death

## Abstract

Here, we identify iPLA2β as a critical regulator for p53-driven ferroptosis upon reactive oxygen species (ROS)-induced stress. The calcium-independent phospholipase iPLA2β is known to cleave acyl tails from the glycerol backbone of lipids and release oxidized fatty acids from phospholipids. We found that iPLA2β-mediated detoxification of peroxidized lipids is sufficient to suppress p53-driven ferroptosis upon ROS-induced stress, even in GPX4-null cells. Moreover, iPLA2β is overexpressed in human cancers; inhibition of endogenous iPLA2β sensitizes tumor cells to p53-driven ferroptosis and promotes p53-dependent tumor suppression in xenograft mouse models. These results demonstrate that iPLA2β acts as a major ferroptosis repressor in a GPX4-independent manner. Notably, unlike GPX4, loss of iPLA2β has no obvious effect on normal development or cell viability in normal tissues but iPLA2β plays an essential role in regulating ferroptosis upon ROS-induced stress. Thus, our study suggests that iPLA2β is a promising therapeutic target for activating ferroptosis-mediated tumor suppression without serious toxicity concerns.

## Abstract (web summary)

p53 is able to induce ferroptosis in response to reactive oxygen species (ROS)-induced stress and suppresses tumour growth. Here, the authors show that iPLA2β suppresses p53-medated ferroptosis by cleaving and detoxifying peroxidized lipids and that this is independent of canonical ferroptosis regulator GPX4.

## Introduction

Inactivation of the p53 tumor suppressor is a pivotal event in the formation of most human cancers. p53 plays a central role by acting as a transcription factor to modulate various types of cellular processes to suppress cancer development^1,2^. Among the multiple layers of mechanisms controlling p53 function, negative regulators in modulating p53 responses such as Mdm2/Mdmx oncoproteins are particularly interesting as these factors act as potential targets for activating p53 function in cancer therapy^3,4^. The exquisite regulation of p53 functions is of vital importance for cell fate decisions. Although the classic activities of p53 including cell cycle arrest, apoptosis, and senescence are well accepted as the major mechanisms in tumor suppression, recent studies suggest that unconventional mechanisms also contribute significantly to tumor suppression^5,6^. We and others recently found that p53 plays an important role in modulating ferroptotic cell death responses in cancer cells through its metabolic targets^7–11^.

Ferroptosis is an iron-dependent form of non-apoptotic cell death induced by excess accumulation of peroxidized phopholipids, generated through oxidation of the polyunsaturated fatty acyl (PUFA) moieties of membrane phospholipids^12–14^. Ferroptosis under normal homeostasis conditions is tightly controlled by glutathione peroxidase 4 (GPX4), which converts lipid hydroperoxides into non-toxic lipid alcohols^15,16^. Recent studies indicate that ferroptosis, like apoptosis acts as a new tool for suppressing tumor growth; inhibitors of GPX4 have been proposed as promising drugs in cancer therapy^17–19^. Ferroptosis induced by GPX4 inhibition is dependent on the activity of acyl-CoA synthetase long-chain family member 4 (ACSL4), which incorporates free fatty acids such as PUFAs into phospholipids (PL-PUFAs)^20,21^. Thus, the cells lacking ACSL4 expression are resistant to ferroptosis induced by GPX4 inhibitors^20^. We and others found that the ability of p53 to induce ferroptosis significantly contributes to its tumor suppression activity^7,8,22^. Notably, p53-mediated ferroptosis acts mainly through the ALOX12 lipoxygenase upon reactive oxygen species (ROS)-induced stress^11^. The ALOX12 gene resides on human chromosome 17p13.1 at a common position with frequent monoallelic deletion in human cancers. Indeed, loss of one ALOX12 allele is sufficient to accelerate tumorigenesis by abrogating p53-mediated ferroptosis and tumor suppression in mouse lymphoma models. Surprisingly, however, ALOX12 is dispensable for ferroptosis induced by GPX4 inhibitors, whereas ACSL4 is not required for p53-mediated ferroptosis upon ROS-induced stress^11^. These studies suggest that p53 promotes ferroptosis through a distinct GPX4-independent pathway critically for its tumor suppression. Moreover, it remains unclear whether any GPX4-like factor is involved in regulating p53-dependent ferroptosis and tumor suppression. Here, we identify iPLA2β as a major suppressor of p53-mediated ferroptosis in the cells subjected to ROS-induced stress. We demonstrate that iPLA2β-mediated detoxification of peroxidized lipids is sufficient to suppress p53-driven ferroptosis in a GPX4-independent manner. Moreover, depletion of endogenous iPLA2β sensitizes tumor cells to ROS-induced ferroptosis and enhances p53-dependent tumor growth suppression. Thus, our results indicate that iPLA2β-mediated lipid detoxification is critically involved in suppressing p53-mediated ferroptosis and tumor suppression and suggest that iPLA2β is a promising target for activating ferroptosis in human cancers.

## Results

### p53-Driven ferroptosis is induced in a GPX4-independent manner

**Fig. 1:**
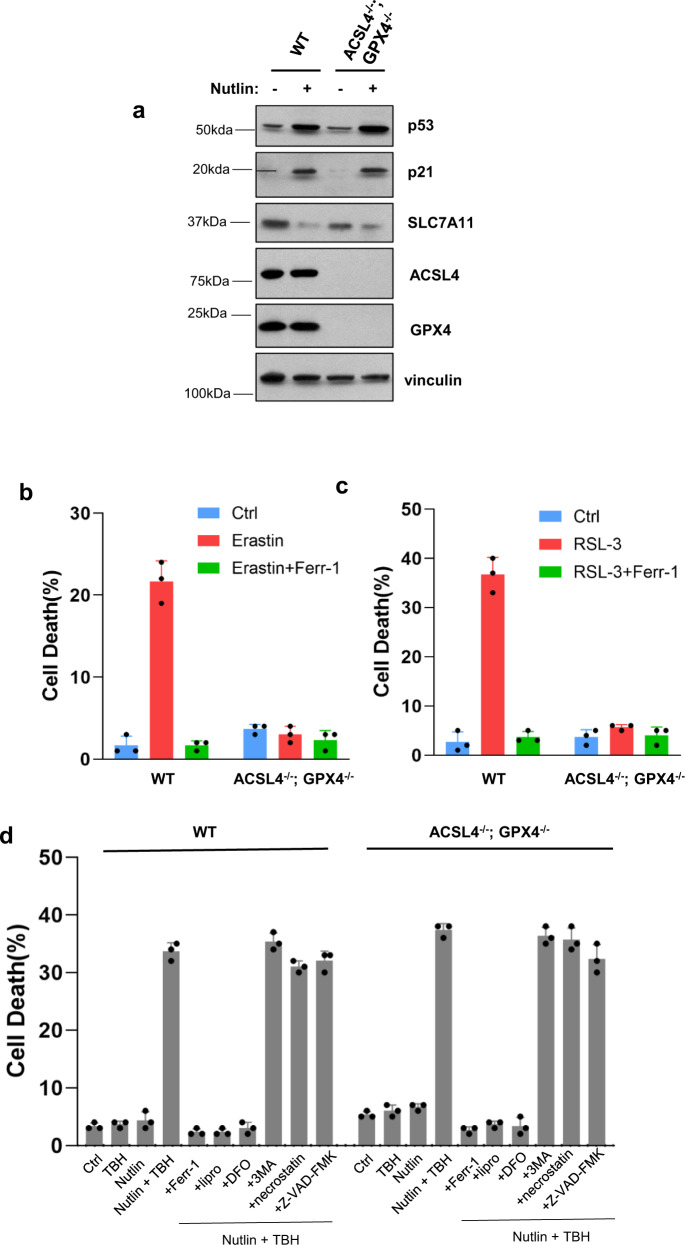
p53-Mediated ferroptosis is GPX4-independent upon ROS-induced stress. **a** Western blot analysis of U2OS ACSL4^−/−^; GPX4^−/−^ cells treated with Nutlin (10 μM) as indicated for 48 h. The experiments were repeated twice, independently, with similar results. **b** U2OS ACSL4^−/−^; GPX4^−/−^ cells were treated with Erastin (40 μM) and Ferr-1 (2 μM) as indicated for 12 h. **c** U2OS ACSL4^−/−^; GPX4^−/−^ cells were treated with RSL-3 (2 μM) and Ferr-1 (2 μM) as indicated for 12 h. **d** U2OS control, ACSL4^−/−^; GPX4^−/−^ cells pre-incubated with Nutlin (10 μM) for 24 h were treated with TBH (250 μM), Nutlin (10 μM), Ferr-1 (2 μM), Lipro-1 (2 μM), 3-MA (2 mM), necrostatin-1 (10 μg/mL), and Z-VAD-FMK (10 μg/mL) as indicated, for 8 h. 3-MA, 3-methylademine. **b**–**d** Error bars are mean ± s.d., *n* = 3 biologically independent experiments. Source data are provided as a Source Data file.

We previously established that p53-dependent ferroptosis can be induced in the presence of elevated levels of ROS, generated by *tert*-Butyl hydroperoxide (TBH) without common ferroptosis inducers such as erastin or GPX4 inhbitors^7^. Although no significant cell death was observed under low levels of TBH treatment in human cancer cells, the combination of TBH treatment and p53 activation (e.g., Nutlin treatment) is able to effectively induce high levels of ferroptosis, even in ACSL4-null cells^11^. Since ACSL4 is essential for ferroptosis induced by GPX4 inhibitors^20,21^, it is likely that p53-mediated ferroptotic response is independent of GPX4 function. However, this notion cannot be directly tested since GPX4-null cells do not survive under normal conditions unless ACSL4 is co-deleted. To address whether p53-mediated ferroptosis can be induced independent of GPX4 function, we generated ACSL4/GPX4 double-knockout derivatives of the human osteosarcoma cell line U2OS. As shown in Fig. 1a, the levels of p53 were unaffected by loss of ACSL4 and GPX4 and, p53-mediated transcription activation of p21 or p53-mediated repression of SLC7A11 remained intact. Also, given their lack of ACSL4, these cells are resistant to ferroptosis induced by either erastin (Fig. 1b) or the GPX4 inhibitor RSL−3 (Fig. 1c). However, ferroptotic cell death was readily observed when ACSL4/GPX4-null cells were exposed to both the ROS generator TBH and the p53 activator Nutlin (Fig. 1d and Supplementary Fig. 1a). As expected, the levels of ferroptosis were largely abrogated when isogenic p53-null, or ALOX12-null U2OS cells were subjected to the same conditions (Supplementary Fig. 1a, b). Similar results were also obtained with independent ACSL4/GPX4-null clones (Supplementary Fig. 2a, b). Moreover, these p53-mediated responses were specifically blocked by known ferroptosis inhibitors (e.g., Ferr−1, DFO, and Lipro-1) but not by inhibitors of other cell death pathways, such as apoptosis, autophagy or necroptosis (Fig. 1d). Similar results were reproduced when an independent cell death assay was used by monitoring cellular ATP levels (Supplementary Fig. 2c).

**Fig. 2:**
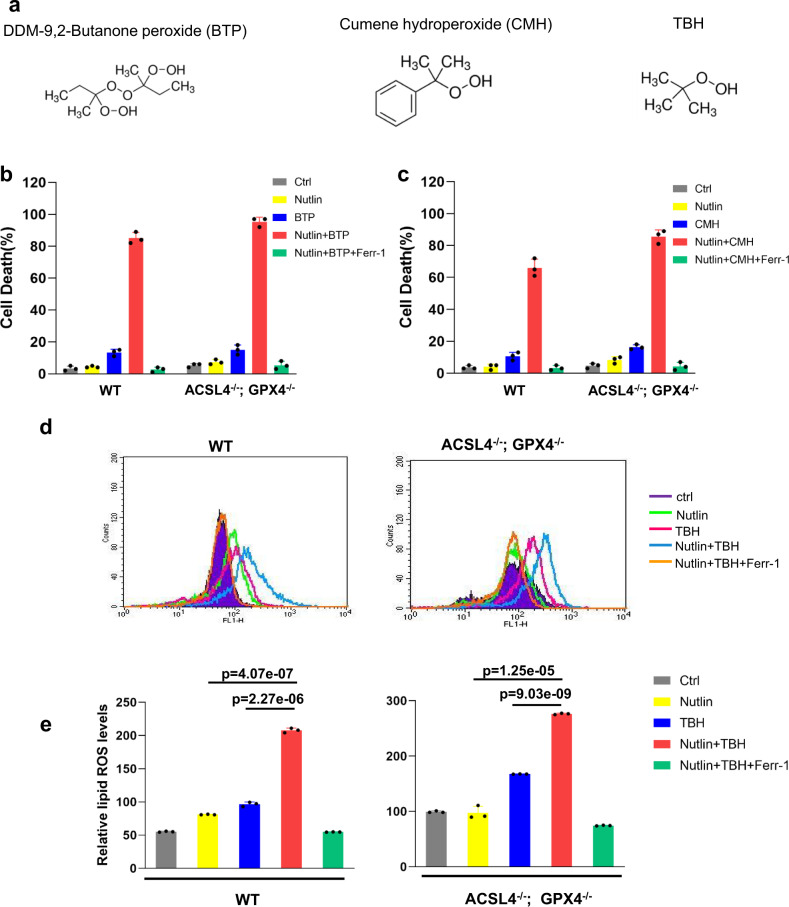
BTP or CMH has similar effects as TBH does. **a** Variant compounds having similar functional group with TBH. **b** U2OS control, ACSL4^−/−^; GPX4^−/−^ cells pre-incubated with Nutlin (10 μM) for 12 h were treated with BTP (300 μM), Nutlin (10 μM), and Ferr-1 (2 μM) as indicated for 8 h. BTP, butanone peroxide. **c** U2OS control, ACSL4^−/−^; GPX4^−/−^ cells pre-incubated with Nutlin (10 μM) for 12 h were treated with CMH (200 μM), Nutlin (10 μM), and Ferr-1 (2 μM) as indicated for 8 h. CMH, cumene hydroperoxide. **d** Lipid peroxidation levels of U2OS control, ACSL4^−/−^; GPX4^−/−^ cells pre-incubated with Nutlin (10 μM) for 12 h were treated with TBH (300 μM), Nutlin (10 μM), and Ferr-1 (2 μM) as indicated for 8 h. **e** Quantification of lipid peroxidation levels is shown as in (**d**). **b**, **c**, **e** Error bars are mean ± s.d., *n* = 3 independent repeats. **e** All *p* values were calculated using two-tailed unpaired Student’s *t*-test. Detailed statistical tests are described in the ‘Methods’. For WT, *p* = 0.000000227, TBH versus Nutlin + TBH; *p* = 0.0000000407, Nutlin versus Nutlin + TBH. For ACSL4^−/−^; GPX4^−/−^, *p* = 0.000000000903, TBH versus Nutlin + TBH; *p* = 0.00000125, Nutlin versus Nutlin + TBH. Source data are provided as a Source Data file.

Next, we examined whether p53-mediated ferroptosis can be elicited by other ROS-generating compounds, such as DDM−9,2-butanone peroxide (BTP) and cumene hydroperoxide (CMH) with very similar chemical structures compared to TBH (Fig. 2a). Indeed, both compounds readily induced high levels of ferroptosis in both parental and ACSL4/GPX4-null U2OS cells under the same conditions (Fig. 2b, c). Moreover, elevated levels of endogenous lipid peroxidation, a key marker of ferroptosis, were observed by flow cytometry with C11-BODIPY staining when either parental or ACSL4/GPX4-null cells were treated with both Nutlin and TBH (Fig. 2d, e). Taken together, these data demonstrate that p53-driven ferroptosis upon high levels of ROS is induced in a GPX4-independent manner.

### iPLA2β is differentially regulated by p53 upon different levels of stress

Under normal homeostasis conditions, GPX4 protects cells from ferroptosis by converting oxidized phospholipids into non-toxic lipid alcohols^15–17^. However, our results indicate that, under conditions of oxidative stress, p53-mediated ferroptosis is independent of GPX4 activity. Thus, we considered whether other modes of lipid detoxification are involved in regulating p53-mediated ferroptosis. Phospholipases can cleave the glycerol backbone of phospholipids to yield free fatty acids and lysophospholipids^23^. Moreover, recent studies have shown that the calcium-independent phospholipase iPLA2γ is able to hydrolyze oxidized cardiolipin (CL), an inner mitochondrial membrane phospholipid and, subsequently release oxidized fatty acids from cardiolipin^24–26^. Interestingly, upon analysis of our RNA-Seq data, we found that the mRNA levels of iPLA2β, another member of the calcium-independent phospholipase A2 family, were induced by p53 activation.

**Fig. 3:**
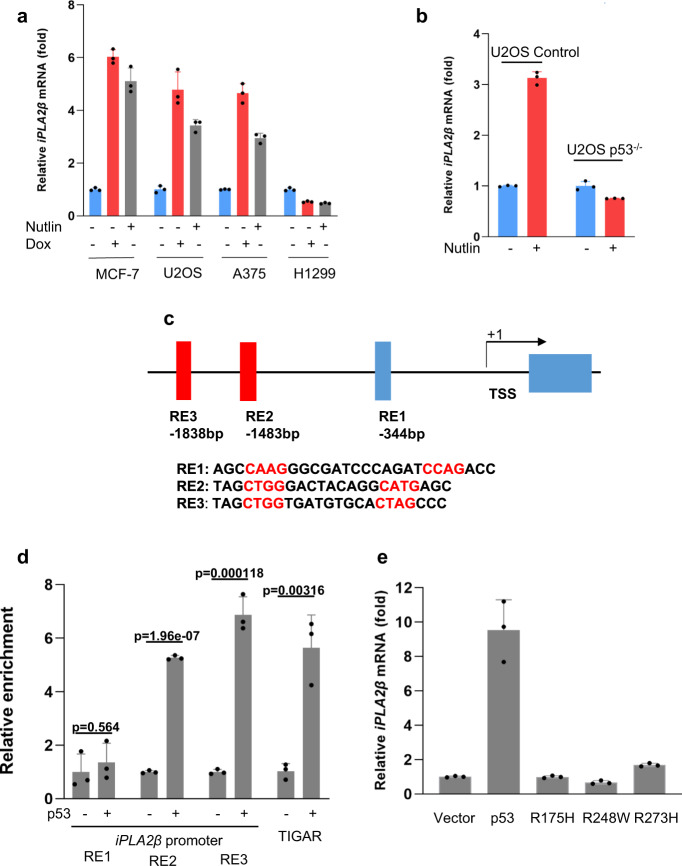
Regulation of iPLA2β during p53-mediated stress responses. **a** qPCR analysis of mRNA levels of *iPLA2β* in the MCF-7, U2OS, A375, and H1299 cells treated with 0.2 μg/mL doxorubicin or 10 μM Nutlin for 24 h. **b** qPCR analysis of mRNA levels of *iPLA2β* in the U2OS CRISPR control versus p53^−/−^ cells treated with 10 μM Nutlin for 24 h. **c** Schematic representation of the promoter region in the human *iPLA2β* gene. The p53-binding sites upstream of the first exon are indicated as responsive elements (RE). TSS, transcription start site. **d** ChIP-qPCR was performed in H1299 cells transfected with empty vector or p53. *p* values were calculated using two-sided unpaired Student’s *t*-test. Detailed statistical tests are described in the ‘Methods’. *p* = 0.564 for RE1; *p* = 0.0000000196 for RE2; *p* = 0.000118 for RE3; and *p* = 0.00316 for TIGAR. **e** H1299 cells were transfected with empty vector, wild-type p53 or mutants (R175H, R273H, and R248W), and *iPLA2β* mRNA levels were analyzed by qRT-PCR. **a**, **b**, **d**, **e** Error bars are mean ± s.d., *n* = 3 biologically independent experiments. Source data are provided as a Source Data file.

To ascertain whether the iPLA2β gene is indeed regulated by p53, we examined iPLA2β expression in response to p53 activation by either Nutlin or doxorubicin, a common DNA damage reagent. iPLA2β mRNA levels were modestly but significantly upregulated by treatment with either Nutlin or doxorubicin in cell lines that express wild-type p53 (U2OS, MCF7, A375, A549 cells), but not in the p53-null line H1299 (Fig. 3a and Supplementary Fig. 3). To confirm that iPLA2β transcription is dependent on p53, we also evaluated U2OS sublines generated by CRISPR-mediated inactivation of the p53 gene. As shown in Fig. 3b, the mRNA levels of iPLA2β were induced by Nutlin treatment in parental, but not p53-knockout, cells. Furthermore, the promoter region of the human iPLA2β gene contains three potential sites (RE1, RE2, and RE3) that match the consensus p53-binding sequence (Fig. 3c). Indeed, chromatin immunoprecipitation (ChIP) analysis with a p53-specific antibody revealed significant recruitment of p53 to RE2 and RE3, but not RE1, at levels comparable to those observed at the promoter of TIGAR^27^, a well-known metabolic target of p53 (Fig. 3d). Of note, iPLA2β mRNA expression was readily induced when p53-null H1299 cells were reconstituted by transient transfection with wild-type p53, but not with three tumor-derived DNA-binding defective p53 mutants (R175H, R273H, and R248W) (Fig. 3e).

**Fig. 4:**
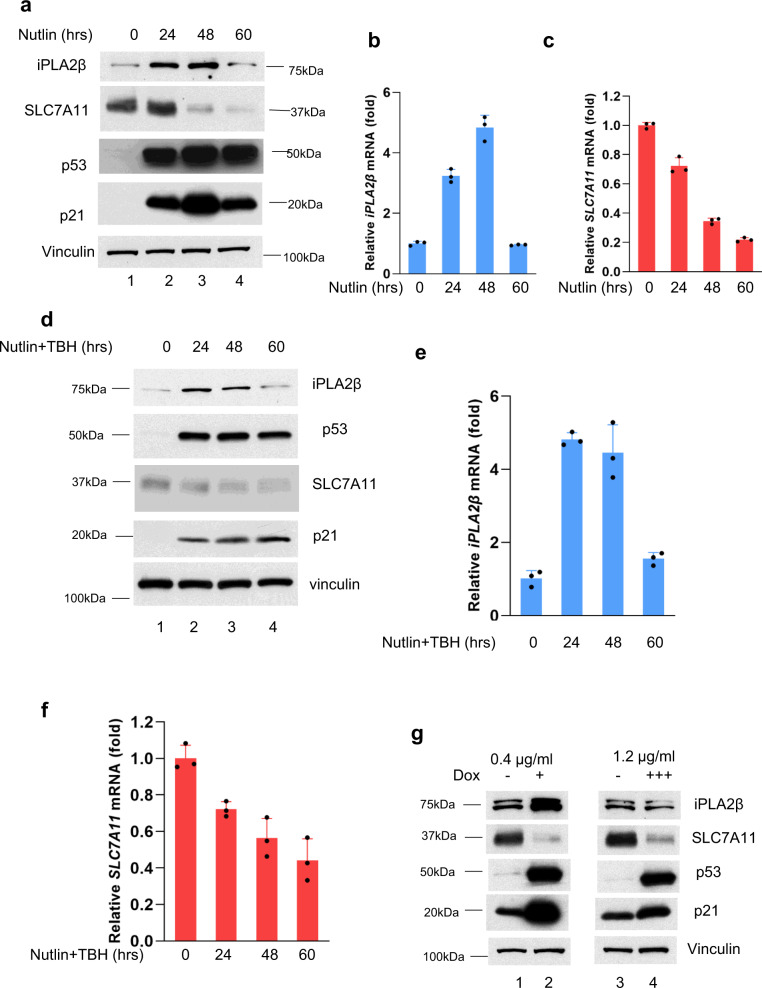
Differential effects on iPLA2β mediated by p53 depends on stress duration and intensity. **a** Western blot analysis of extracts of U2OS cells with different time of 10 μM Nutlin treatment. The experiments were repeated twice, independently, with similar results. **b** qPCR analysis of mRNA levels of *iPLA2β* for the above cells. **c** qPCR analysis of mRNA levels of *SLC7A11* for the same cells as (**a**). **d** Western blot analysis of extracts of U2OS cells with different time of 10 μM Nutlin + TBH treatment. The experiments were repeated twice, independently, with similar results. **e** qPCR analysis of mRNA levels of *iPLA2β* for the same cells as (**d**). **f** qPCR analysis of mRNA levels of *SLC7A11* for the same cells as (**d**). **g** Western blot analysis of extracts of HCT116 cells with low dose (lanes 1, 2) or high dose of doxorubicin (lanes 3, 4) as indicated for 30 h. The experiments were repeated twice, independently, with similar results. **b**, **c**, **e**, **f** Error bars are mean ± s.d., n = 3 biologically independent experiments. Source data are provided as a Source Data file.

To further understand the nature of this regulation, we examined the levels of iPLA2β under different lengths or different dosages of the same treatments during p53-mediated responses. Notably, the levels of iPLA2β were induced at earlier time points but the activation was completely abrogated at a later time point after Nutlin treatment (Fig. 4a, b); conversely, p53-mediated regulation of p21 were observed at all different of time points (Fig. 4a). Since our previous studies showed that p53-mediated regulation of SLC7A11 is critical for ferroptosis^7,11^, we also monitored the levels of SLC7A11. Indeed, downregulation of SLC7A11 by p53 remained intact at all different of time points (Fig. 4a–c). Similar data were also obtained when the cells were treated with both Nutlin and TBH (Fig. 4d–f), as p53-mediated ferroptosis was examined upon the treatment of both Nutlin and TBH.

Moreover, the levels of iPLA2β were induced upon a low dose of the DNA damage reagent but the activation was largely abrogated with a high dose of the same reagent in HCT116 cells, whereas p53-mediated regulation of SLC7A11 and p21 remained intact under both conditions (Fig. 4g). In addition to HCT116 cells, we also performed the same treatment in both U2OS cells and A549 cells. Indeed, similar results were obtained to support the same conclusion (Supplementary Fig. 4a–i). Taken together, although the precise mechanism of p53-mediated transactivation of iPLA2β needs further elucidation, these data suggest that iPLA2β is a direct target of p53 but p53-mediated regulation of iPLA2β is very sensitive to the conditions of the treatment. Indeed, iPLA2β is activated by p53 at the early stage of stress responses or under low levels of stress but this activation is diminished at the late stage of stress responses or under high levels of stress.

### Depletion of endogenous iPLA2β sensitizes tumor cells to ROS-induced ferroptosis and enhances p53-dependent tumor growth suppression in xenograft mouse models

**Fig. 5:**
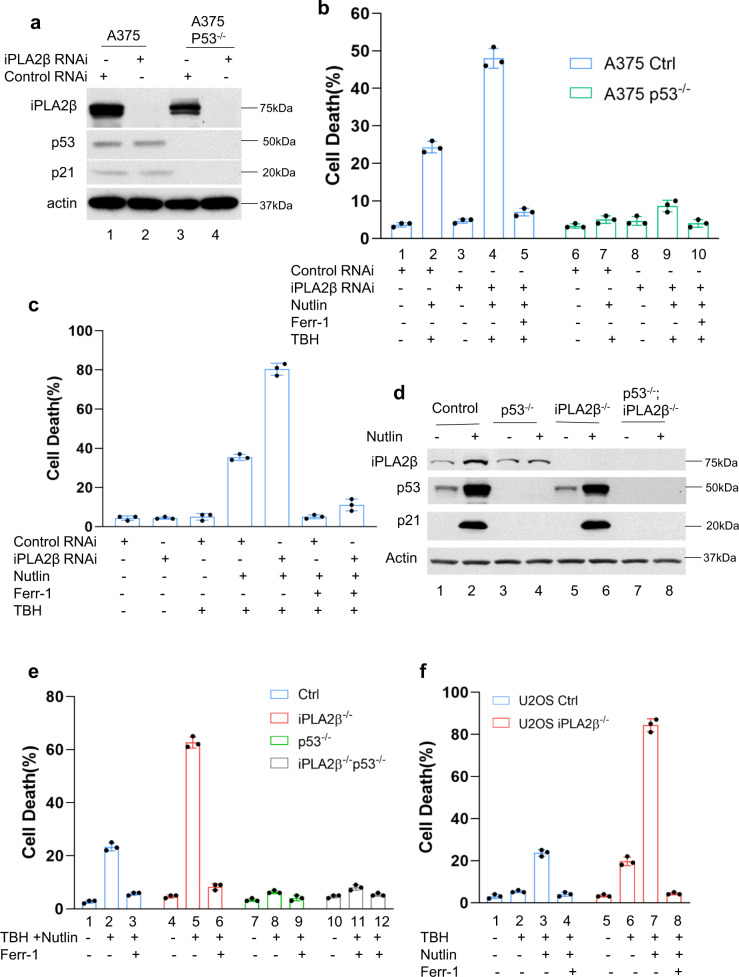
iPLA2β acts as a major suppressor of p53-mediated ferroptosis. **a** Western blot analysis of extracts of A375 CRISPR control (lanes 1, 2) versus p53 ^−/−^ cells (lanes 3, 4) treated with control RNAi (lanes 1, 3) or iPLA2β RNAi (lanes 2, 4) by the antibodies to iPLA2β, p53, p21, or actin. The experiments were repeated twice, independently, with similar results. **b** Quantification of ROS-induced ferroptotic cell death. The same cells in (**a**) were pre-incubated with 10 μM Nutlin for 24 h, then treated with 120 μM TBH and 10 μM Nutlin (error bars, s.d. from three independent samples). **c** Quantification of ferroptotic cell death-mediated by ROS. After iPLA2β RNAi treatment, MCF-7 cells were pre-incubated with 10 μM Nutlin for 24 h, then treated with 80 μM TBH and 10 μM Nutlin (error bars, s.d. from three independent samples). **d** Western blot analysis of extracts of A375 CRISPR control (lanes 1, 2), p53^−/−^ cells (lanes 3, 4), iPLA2β^−/−^ cells (lanes 5, 6), or p53^−/−^; iPLA2β^−/−^ cells treated with Nutlin 10 μM for 24 h (lanes 2, 4, 6, 8) versus control (lanes 1,3, 5, 7) by the antibodies to iPLA2β, p53, p21, or actin. The experiments were repeated twice, independently, with similar results. **e** Quantification of cell death in the same cells as (**d**) with additional of 150 μm TBH as indicated. Error bars are mean ± s.d., *n* = 3 biologically independent experiments. **f** Quantification of ROS-mediated ferroptotic cell death. U2OS CRISPR control versus iPLA2β^−/−^ cells pre-incubated with Nutlin (10 μM) for 24 h were treated with TBH (250 μM), Nutlin (10 μM) and Ferr-1 (2 μM) as indicated (error bars, s.d. from three independent replicates). Source data are provided as a Source Data file.

**Fig. 6:**
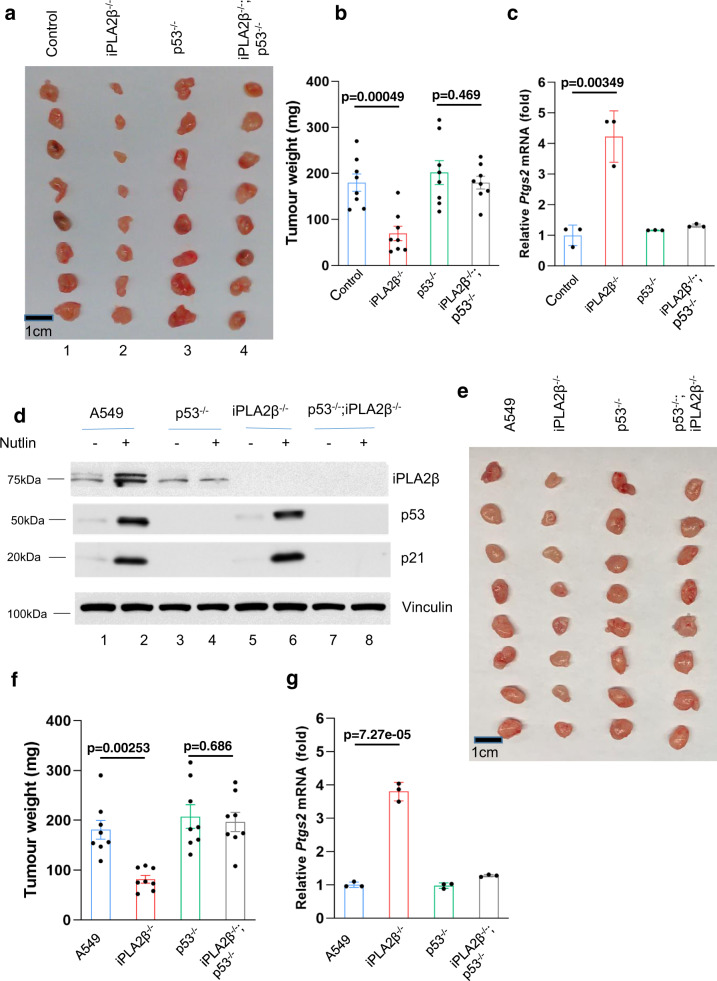
iPLA2β acts as a major suppressor of p53-mediated tumor suppressor. **a** Xenograft tumors from A375 CRISPR control, iPLA2β^−/−^, p53^−/−^ or p53^−/−^; iPLA2β^−/−^ cells as indicated. **b** Tumor weights were determined from (**a**) (error bars, from eight tumors). The experiments were repeated twice independently with similar results and representative data were shown. *p* = 0.00049 for control versus iPLA2β^−/−^; *p* = 0.469 for p53^−/−^ versus iPLA2β^−/−^; p53^−/−^. **c** qPCR of *Ptgs2* mRNA from tumors harvested in (**a**). *p* = 0.00349 for control versus iPLA2β^−/−^. **d** A549 cells (Lanes 1, 2), p53^−/−^ cells (lanes 3, 4), iPLA2β^−/−^ cells (lanes 5, 6), or p53^−/−^; iPLA2β^−/−^ cells (lanes 7, 8) treated with Nutlin 10 µM for 24 h (lanes 2, 4, 6, 8) versus control (lanes 1, 3, 5, 7) by the antibodies to iPLA2β, p53, p21or actin. The experiments were repeated twice, independently, with similar results. **e** Xenograft tumors from A549 CRISPR control, iPLA2β^−/−^, p53^−/−^ or p53^−/−^; iPLA2β^−/−^ cells as indicated. **f** Tumor weights were determined from (**e**) (error bars, from eight tumors). The experiments were repeated twice independently with similar results and representative data were shown. *p* = 0.00253 for A549 versus iPLA2β^−/−^; *p* = 0.686 for p53^−/−^ versus iPLA2β^−/−^; p53^−/−^. **g** qPCR of *Ptgs2* mRNA from tumors harvested in (**e**). *p* = 0.00000727 for A549 versus iPLA2β^−/−^. **b**, **f** Error bars are mean ± SEM. **c**, **g** Error bars are mean ± s.d., **b**–**c**, **f**–**g** *n* = 8 biologically independent samples. All *p* values were calculated using two-tailed unpaired Student’s *t*-test. Detailed statistical tests are described in the ‘Methods’. Source data are provided as a Source Data file.

To investigate the role of iPLA2β in modulating p53 functions, we first examined whether RNAi-mediated knockdown of endogenous iPLA2β affects p53-dependent ferroptosis in human melanoma A375 cells. To this end, both A375 and isogenic p53-null A375 cells were transfected with siRNAs (Dharmacon SMART-pools) specific for iPLA2β. Western blot analysis revealed that siRNA-mediated iPLA2β depletion did not affect the expression levels of p53 or its transcriptional target p21 (Fig. 5a). As expected, p53-mediated ferroptosis was readily induced in TBH-treated cells transfected with control siRNAs (Fig. 5b). However, the levels of ferroptosis were significantly increased by iPLA2β depletion in wild-type p53 (Fig. 5b), but no significant effect was observed in p53-null cells under the same conditions (Fig. 5b), suggesting that iPLA2β can suppress p53-mediated ferroptosis. Similar results were also obtained in U2OS cells and isogenic p53-null U2OS cells by the same methods (Supplementary Fig. 5a, b). Previously, we showed that MCF7 human breast carcinoma cells are susceptible to p53-mediated ferroptosis^11^ but, owing to a lack of ACSL4 expression, they are resistant to ferroptosis induced by either direct (e.g., RSL−3) or indirect (erastin) GPX4 inhibitors^11,20^. As shown in Fig. 5c, iPLA2β knockdown significantly enhanced p53-mediated ferroptosis in MCF7 cells (the levels of iPLA2β were showed in Supplementary Fig. 6a). To further validate the role of iPLA2β in p53-mediated ferroptosis, we used CRISPR/Cas9 technology to inactivate the iPLA2β gene in A375 cells. Consistent with the above data, loss of iPLA2β expression had no obvious effect on p53 levels and p53 transcriptional function (Fig. 5d) but p53-mediated ferroptosis was significantly elevated in iPLA2β-null cells at different time points (Fig. 5e and Supplementary Fig. 6b, c). Likewise, p53-mediated ferroptosis was markedly enhanced by iPLA2β depletion in U2OS cells (Fig. 6f and Supplementary Fig. 6d). Collectively, these data demonstrate that loss of iPLA2β expression significantly sensitizes these cancer cells to p53-dependent ferroptosis upon ROS-induced stress.

To elucidate the physiological significance of iPLA2β in ferroptosis regulation, we tested whether loss of iPLA2β expression affects tumor growth in xenograft tumor models. Indeed, the growth of human melanoma A375 xenograft tumors was dramatically reduced upon inactivation of iPLA2β expression (2 vs. 1, Fig. 6a, b) in xenograft tumor growth assays; however, the tumor suppressive effects induced by loss of iPLA2β expression were largely abrogated when isogenic p53-null A375 cells were used (4 vs. 3, Fig. 6a, b). Notably, upregulation of *Ptgs2*, a marker of ferroptosis, was observed in iPLA2β^−/−^ tumor samples but not in iPLA2β^−/−^/p53^−/−^ samples (Fig. 6c). Finally, to further validate these observations, we performed a series of the similar experiments described above in human lung carcinoma A549 cells. Again, loss of iPLA2β significantly enhanced p53-mediated ferroptosis (Fig. 6d and Supplementary Fig. 7). More importantly, inactivation of iPLA2β also promoted the tumor suppressive effects in p53-WT cells but not isogenic p53-null cells (Fig. 6e–g). Taken together, these data demonstrate that the inhibition of iPLA2β expression significantly enhances p53-mediated ferroptosis and that activation of ferroptosis by loss of iPLA2β, at least, in part contributes to p53-dependent tumor growth suppression in vivo.

### iPLA2β downregulates the levels of peroxidized membrane lipids induced by ROS exposure and effectively suppresses p53-driven ferroptosis

**Fig. 7:**
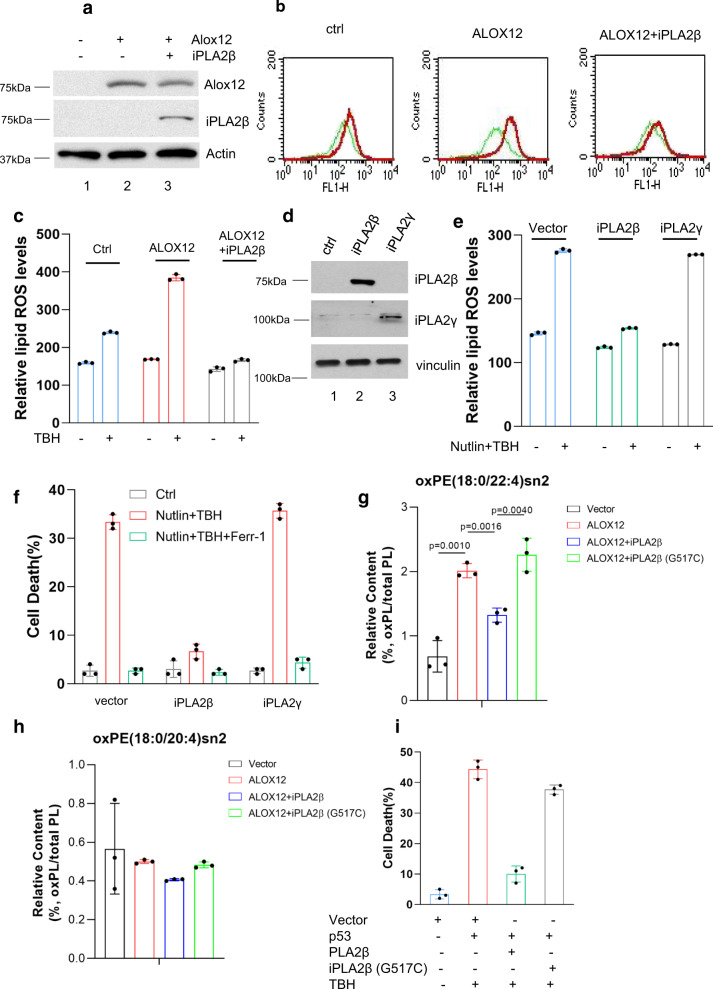
Mechanistic insights into iPLA2β–mediated suppression of lipid peroxidation and ferroptosis induced by p53 and ROS stress. **a** Western blot analysis of U2OS ACSL4^−/−^; GPX4^−/−^ cells transfected with AlOX12 or iPLA2β vector. The experiments were repeated twice, independently, with similar results. **b** Lipid peroxidation levels of U2OS ACSL4^−/−^; GPX4^−/−^ cells transfected with ALOX12 or iPLA2β vector were treated with TBH (300 μM) and Ferr-1 (2 μM) as indicated for 8 h. **c** Quantification of lipid peroxidation levels as shown in (**b**). Error bars are mean ± s.d., *n* = 3 independent experiments. **d** Western blot analysis of U2OS ACSL4^−/−^; GPX4^−/−^ cells transfected with iPLA2β or iPLA2γ vector. The experiments were repeated twice, independently, with similar results. **e** Quantification of Lipid peroxidation levels. U2OS ACSL4^−/−^; GPX4^−/−^ cells with overexpression of iPLA2β or iPLA2γ were pre-incubated with Nutlin (10 μM) for 12 h, then treated with TBH (300 μM) and Nutlin (10 μM) as indicated for 8 h. **f** Quantification of cell death. U2OS ACSL4^−/−^; GPX4^−/−^ cells with overexpression of iPLA2β or iPLA2γ were pre-incubated with Nutlin (10 μM) for 24 h, then treated with TBH (250 μM) and Nutlin (10 μM) as indicated. **g** Relative content of oxPE (18:0/22:4)sn2 in H1299 cells transfected with alox12, or/and iPLA2β (PE, phosphatidylethanolamines). *p* values were calculated using two-tailed unpaired Student’s *t*-test. Detailed statistical tests are described in the ‘Methods’. **h** Relative content of oxPE (18:0/20:4)sn2 in H1299 cells transfected ALOX12, or/and iPLA2β. **i** Quantification of cell death in H1299 cells transfected with vector, p53, iPLA2β WT or G517C, and additional TBH treatment. **e**–**i** Error bars are mean ± s.d., *n* = 3 biologically independent experiments. Source data are provided as a Source Data file.

Recent studies indicate that oxidized PUFA-containing phospholipids, but not free oxidized PUFAs, act as key executioners of ferroptosis^21^. The calcium-independent phospholipase family enzymes are known for their abilities to cleave acyl tails from the glycerol backbone of lipids and release oxidized fatty acids such as PUFAs, from membrane phospholipids^23–26^. Thus, it is very likely that iPLA2β suppresses ferroptosis by cleaving peroxidized lipids for detoxification. Since ALOX12 is required for p53-mediated ferroptosis upon ROS stress^11^, we first examined whether ALOX12 expression can promote lipid peroxidation upon ROS-induced stress and whether the levels of the oxidized phospholipids induced by ALOX12 are suppressed by iPLA2β. To this end, U2OS cells were transfected with expression vectors encoding ALOX12 alone or both ALOX12 and iPLA2β (Fig. 7a), stained with C11-BODIPY, and analyzed by flow cytometry to assess levels of endogenous lipid peroxidation from cell membrane. As shown in Fig. 7b, c, peroxidized lipid levels were markedly increased by ALOX12 expression; however, these elevated levels were effectively reduced upon co-expression of iPLA2β. As expected, iPLA2β expression also reduced the levels of ferroptosis induced by ALOX12 expression (Supplementary Fig. 8). These data indicate that iPLA2β can suppress ferroptosis by abrogating ALOX12-induced lipid peroxidation.

Since p53-mediated ferroptosis is independent of GPX4 (Fig. 1d), we examined whether iPLA2β can also modulate p53-mediated ferroptosis in GPX4-null cells. To this end, we transfected ACSL4/GPX4-null U2OS cells with expression vectors encoding either iPLA2β or, iPLA2γ as an additional control (Fig. 7d), and analyzed lipid peroxidation levels by flow cytometry with C11-BODIPY staining. As shown in Fig. 7e, the elevated levels of lipid peroxidation induced by p53 activation under ROS stress in ACSL4/GPX4-null cells (I) were significantly reduced by expression of iPLA2β (II), but not by iPLA2γ expression (III, Fig. 7e). Moreover, p53-mediated ferroptosis was completely abrogated by overexpression of iPLA2β but not by iPLA2γ expression in these GPX4-null cells (Fig. 7f). Thus, iPLA2β suppresses p53-mediated ferroptosis by reducing the levels of lipid peroxidation in a GPX4-independent manner.

Next, we performed targeted lipidomics to monitor the levels of peroxidized lipids regulated by iPLA2β. Indeed, the levels of the oxidized phosphatidylethanolamine (PE) and phosphatidylcholine (PC) (oxPE (18:0/22:4)sn2 or oxPC (18:0/20:4)sn2) were significantly induced upon ALOX12 expression, indicating that ALOX12 is critical for generating oxidized PE and oxidized PC in human cancer cells (Fig. 7g, h and Supplementary Fig. 9a–c). However, the increased levels of the oxidized phosphatidylethanolamine (PE) and phosphatidylcholine (PC) (oxPE (18:0/22:4)sn2 or oxPC (18:0/20:4)sn2) were dramatically reduced upon expression of iPLA2β but not the enzymatic defective mutant iPLA2β (G517C) (Fig. 7g, h and Supplementary Fig. 9a, b). Consistent with these observations, this enzymatic defective mutant iPLA2β (G517C) also failed to suppress p53-mediated ferroptosis (Fig. 7i). These data demonstrate that both ALOX12 and iPLA2β play critical roles in directly regulating the levels of the oxidized phosphatidylethanolamine (PE) and phosphatidylcholine (PC) during ferroptotic responses.

**Fig. 8:**
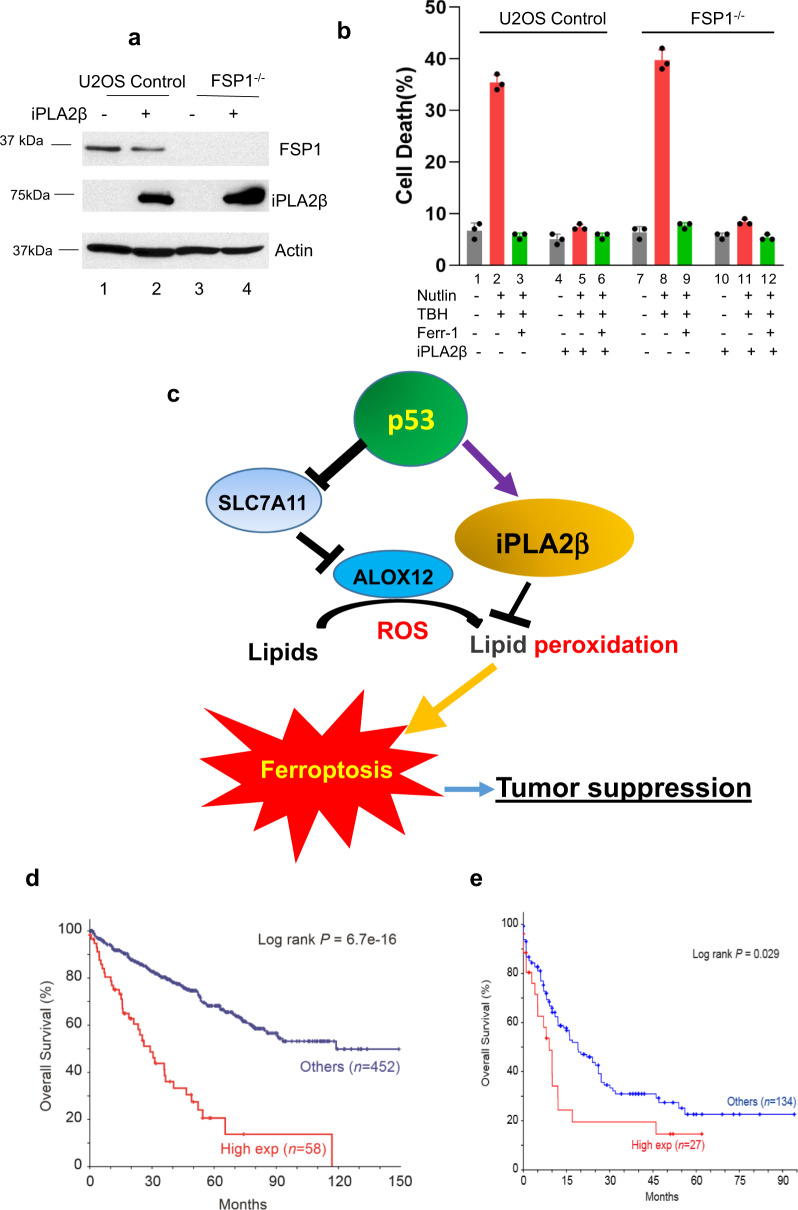
The roles of ALOX12 and iPLA2β in regulating p53-mediated ferroptosis. **a** Western blot analysis of extracts of U2OS CRISPR control (lanes 1, 2) or FSP1^−/−^ cells (lanes 3, 4) with overexpression of iPLA2β-V5 (lanes 2, 4) versus empty vector (lanes 1, 3) by the antibodies to FSP1, V5, or actin. The experiments were repeated twice, independently, with similar results. **b** Quantification of cell death in the same cells as (**a**) treated with 250 μM TBH after pretreatment with Nutlin 10 μM for 24 h. Error bars are mean ± s.d., *n* = 3 biologically independent experiments. **c** A model for the roles of ALOX12 and iPLA2β in regulating p53-mediated ferroptosis. **d** Kaplan-Meier survival curve was generated for overall survival by stratifying patient samples with kidney renal clear cell carcinoma (TCGA, PanCancer Atlas) from cBioPortal based on iPLA2β expression levels. High-expression group was defined as mRNA expression *z*-score > 1 (PLA2G6: EXP > 1). **e** Kaplan-Meier survival curve was generated for overall survival by stratifying patient samples with acute myeloid leukemia (TCGA, PanCancer Atlas) from cBioportal based on iPLA2β expression levels. **d**, **e** *p* values were calculated using two-sided unpaired Student’s *t*-test. Detailed statistical tests are described in the ‘Methods’. Source data are provided as a Source Data file.

Finally, two recent studies have identified FSP1 (ferroptosis suppressor protein 1) as another important regulator for ferroptosis induced by GPX4 inhibitors^28,29^. The FSP1–CoQ_10_–NAD(P)H pathway exists as a parallel system, which co-operates with GPX4 and glutathione to suppress phospholipid peroxidation and ferroptosis. To examine whether iPLA2β-mediated effects on ferroptosis can be induced independent of FSP1 function, we generated FSP1 derivatives of the human osteosarcoma cell line U2OS. As expected, these FSP1-null cells are more sensitive to ferroptosis induced by the GPX4 inhibitor RSL-3 (Supplementary Fig. 10). Nevertheless, p53-mediated ferroptosis was readily induced in FSP1-null cells and more importantly, the cell death was effectively suppressed by iPLA2β in those cells (Fig. 8a, b). Together, these data demonstrate that iPLA2β-mediated effects on ferroptosis are completely independent of GPX4 or FSP1.

## Discussion

Our results indicate that iPLA2β-mediated lipid detoxification is critical for suppressing ROS-induced ferroptosis in cancer cells. By using mouse tumor models, we and others previously showed that p53-mediated ferroptosis acts as a new tumor suppression mechanism independent of the classic mechanisms such as cell cycle arrest, apoptosis, and senescence^7,8,10,22^. Thus, at first glance, it might seem surprising that p53 can activate transcription of the iPLA2β gene, which encodes a potent suppressor of p53-mediated ferroptosis. Yet, in this regard, there are intriguing parallels between the manner in which p53 regulates apoptosis and ferroptosis. Previous studies have established that p53 induces apoptosis under conditions of high stress by transactivation of apoptotic effector genes, such as PUMA and NOXA^30^. However, under low-stress conditions, p53 can instead transactivate expression of the pro-survival genes such as TIGAR, which protects cells from apoptosis^27^. Thus, by differentially regulating both pro- and anti-apoptotic gene, p53 can ensure that apoptosis ensues only when the cell death becomes inevitable (e.g., the damage is unrepairable). Indeed, p53-mediated activation of iPLA2β is only observed at the early stage of stress responses or under low levels of stress but this activation is diminished at the late stage of stress responses or under high levels of stress. Similar to the effects on tumor development by loss of TIGAR^31^, inactivation of iPLA2β enhances the tumor suppressive effects of p53. Thus, like TIGAR, iPLA2β may help cells to survive transient or under low levels of stress; however, under high levels of stress or at the late stage of the stress responses, p53-mediated activation of iPLA2β is abrogated and high levels of ferroptotic responses are designed to effectively eliminate the damaged cells (Fig. 8c).

Although iPLA2β and GPX4 both modulate ferroptosis by detoxifying lipid peroxides, each does so in a biochemically distinct manner: while GPX4 protects cells from ferroptosis by converting oxidized phospholipids into non-toxic lipid alcohols, iPLA2β suppresses ferroptosis by cleaving peroxidized lipids for detoxification. The existence of two distinct lipid repair mechanisms raises interesting questions regarding their different physiological functions. The physiological significance of GPX4 in regulating ferroptosis is best illustrated by the severe phenotypes associated with *Gpx4*-mutant mice^32–35^. For example, while *Gpx4*-null mice undergo early embryonic lethality^32^, studies of conditional *Gpx4*-mutant mice show that GPX4 loss also elicits cell death in multiple adult tissues^33^. Moreover, under normal conditions, most cell lines cannot be successfully cultured in the absence of GPX4^20^. In contrast, we have successfully established iPLA2β^–/–^ cell lines and grew these cells under normal conditions without any difficulty. Moreover, the iPLA2β gene is not required for embryonic development or normal cell homeostasis. Indeed, iPLA2β^–/–^ mice develop normally and grew to maturity^36,37^. Thus, it seems likely that GPX4 and iPLA2β regulate ferroptosis in different physiological settings. On one hand, *Gpx4* may serve as a ‘housekeeping’ gene that controls the levels of lipid peroxidation under conditions of normal homeostasis. On the other hand, iPLA2β is not required for ferroptotic responses under normal homeostasis conditions but is critical for controlling lipid peroxidation levels and ferroptosis during stress responses (Fig. 8c).

The precise mechanism by which iPLA2β-mediated repression of ROS-induced ferroptosis contributes to cancer development needs further elucidation. Through expression profile analysis of iPLA2β from the cancer databases, we found that iPLA2β is overexpressed in many human cancers including kidney renal clear cell carcinoma (RCC) and acute myeloid leukemia (AML) (https://www.cbioportal.org/); more importantly, the cancer patients expressing higher levels of iPLA2β have a significantly shorter overall survival (Fig. 8d, e). Although the p53 gene is frequently mutated (more than 50%) in most of human cancers, the mutation rate of p53 is very low in either RCCs or AMLs (https://p53.iarc.fr). Thus, these data suggest that repression of p53-dependent ferroptosis by iPLA2β may play an important role in tumor development of these cancers. Notably, cancer cells rewire cellular metabolism to meet the energetic and substrate demands of tumor development, but this rewiring also creates metabolic vulnerabilities specific for cancer cells^38^. To fuel their enhanced growth and proliferation, cancer cells undergo metabolic reprogramming, generally accompanied by increased ROS production^38,39^. Thus, the levels of ROS are much higher in cancer cells than in normal cells, making the cancer cells more susceptible to ROS-induced ferroptosis. Given that loss of iPLA2β, in contrast to GPX4, does not affect normal development or cell viability in normal tissues but iPLA2β plays an essential role in ROS-induced ferroptosis in tumor cells, we propose iPLA2β as a more promising therapeutic target than GPX4 for activating ferroptosis in human cancers without causing severe toxicity.

## Methods

### Cell culture and stable lines

U2OS, H1299, A375, MCF7, and A549 cancer lines were ordered from American Type Culture Collection (ATCC) and have been proven to be negative for mycoplasma contamination. All cell lines used in this work were not listed in the ICLAC database. Cells were always cultured in a 37 °C incubator with 5% CO_2_. Media used for cells were supplemented in DMEM with 10% FBS, 100 U/mL penicillin and 100 µg/mL streptomycin (all from Gibco). To obtain iPLA2β, CRISPR-cas9-knockout U2OS cells were generated by transfecting iPLA2β double nickase plasmid (sc-402107-NIC; Santa Cruz). Identical strategies were used to generate p53-knockout A375 cells (p53, sc-416469-NIC), iPLA2β or both p53- and iPLA2β-knockout A375 cells. The cells were selected with puromycin (1 μg/mL) for 4–6 days. iPLA2β, p53-knockout, or both iPLA2β- and p53-knockout single clones were then screened and acquired after continuing to culture 2–3 weeks without selective antibiotics. p53 CRISPR-knockout, alox12-knockout, ACSL4 and GPX4-knockout U2OS cells have been described previously^11^. For FSP1 CRISPR U2OS cells, CRISPR guide RNA (sgRNA) sequences targeting *FSP1* 5′ caccgGAATCGGGAGCTCTGCACG 3′ (22) was synthesized by Invitrogen. Lipofectamine^®^CRISPRMAX were used for transfection following manufacture’s protocol (Invitrogen). Individual clone of U2OS for FSP1^−/−^ was confirmed by western blot.

### Plasmids

Full-length iPLA2β cDNA from Korolev lab (Saint Louis University)^40^, was subcloned into pcDNA3.1/v5-His-Topo vector (Invitrogen). To prepare mutant construct iPLA2β, cDNA sequences corresponding to the region was amplified by PCR from above constructs using QuikChange Site-Directed Mutagenesis Kit (Stratagene) and subcloned into pcDNA3.1/v5-His-Topo vector. GFP-ipla2γ plasmid was generously gifted from Cybulsky lab (McGill University)^41^. p53 WT and its mutant plasmids (R175H, R248W, and R273H) have been described previously^9^.

### Western blotting and antibodies

Protein extracts were blotted by Western according to standard protocols using primary antibodies specific for p53 (sc126, DO-1; Santa Cruz, 1:1000 dilution), iPLA2β (sc376563, D-4, Santa Cruz, 1:200 dilution), MDM2 (SRP2095, Ab5; Millipore, 1:500 dilution), p21 (C-19; Santa Cruz, 1:400 dilution), ACSL4 (sc-271800; Santa Cruz, 1:1000 dilution), GPX4 (ab125066; Abcam, 1:500 dilution), V5 (46-0725; Invitrogen, 1:500 dilution), ALOX12 (sc-365194; Santa Cruz, 1:200 dilution), AMID (FSP1) (sc-377120; Santa Cruz, 1:400 dilution), SLC7A11 (D2M7A) (12691 s; Cell Signaling, 1:500 dilution), Vinculin (V9264; Sigma-Aldrich, 1: 5000 dilution), Actin (A3853; Sigma-Aldrich, 1:5000 dilution), GFP (565271; BD Biosciences, 1:1000 dilution), and TIGAR (sc-166290, Santa Cruz, 1:500 dilution). HRP-conjugated anti-mouse (111-035-146, Jacksonimmuno, 1:500 dilution), and HRP-conjugated anti-rabbit (111-035-045, Jacksonimmuno, 1:500 dilution) secondary antibodies were used.

### Ablation of endogenous iPLA2β by RNAi

Knockdown of iPLA2β protein was performed by transfection of U2OS, A375, and MCF-7 cells with siRNA duplex oligo (ON-TARGET plus SMARTpool: L-00908; Dharmacon) by Lipofectamine 3000 (Invitrogen) for 24 h and then transfected again according to the manufacturer’s protocol.

### RNA extraction and qRT-PCR

Total RNA was isolated using TRIzol (Invitrogen) according to the manufacturer’s protocol. cDNA was reversed by SuperScript^TM^ IV VILO^TM^ Master Mix (Invitrogen). For the qRT-PCR analysis, the following primers were used: human *iPLA2β* forward 5′-GCAATGCTCGGTGCAACAT-3′, *iPLA2β* reverse 5′ - ACACCCCTTCTGAGAGAACTTCA-3′; human *HPRT* forward, TATGGCGACCCGCAGCCCT, *HPRT* reverse, CATCTCGAGCAAGACGTTCAG (also see Supplementary Table 1).

### Chromatin immunoprecipitation assay

The assay was performed as described previously with a little modifications^7^. In short, cells were crosslinked with 1% formaldehyde for 10 min at room temperature and neutralized with a final concentration of 0.125 M glycine. The harvested cells were suspended in cold lysis buffer (10 mM Tris-Cl, pH8.0, 85 mM KCl, 0.25% triton, 0.5% NP40, 5 mM EDTA and proteinase inhibitor mixture). After 10 min incubation at 4 °C, nuclei were harvested and lysed in CHIP buffer (50 mM Tris-HCl pH 8.0, 1% SDS, 5 mM EDTA and protease inhibitor mixture). After sonication, the lysates were centrifuged, the supernatants were added with magnetic beads coated with the specific p53 antibody (full length 393, Santa Cruz) and incubated overnight. Beads were washed seven times with washing buffer (50 mM HEPES, pH7.5, 1 mM EDTA, 500 mM HCl, 0.7% Na-deoxycholate and 1% NP40) and once with TE buffer before the protein–DNA complex was eluted with 0.1 M NaHCO3 and 1% SDS. After reverse crosslinking overnight at 55°C, DNA was extracted and qPCR performed. *iPLA2β* primers: RE1 forward 5′-GCCGGCTCTGTATCTCTCAA-3′, reverse 5′-ACCGAATGACCCTGGGAAGG-3′; RE2 forward 5′-CCTCTGCCTTCTGGGCTTAA-3′, reverse 5′-ACATGGTGAAACCCCATCTC-3′; RE3 forward 5′-GCATCACTGGTCTCTGTCGC-3′, reverse 5′ GTCTAAAATGGGGTTCTGCT-3′. TIGAR primers: forward 5′-CGGCAGGTCTTAGATAGCTT-3′, reverse 5′-GGCAGCCGGCATCAAAAACA-3′ (also see Supplementary Table 1).

### Cell death assay

For cell death assays^7,9,11,42^, cells were trypsinized, collected and stained with trypan blue. The cells were counted with a hemocytometer using the standard protocol, and cells stained blue were considered as dead cells. The cell death was further confirmed by propidium iodide staining followed by FACS analysis. For CellTiter-Glo luminescent assay^43^, 10^4^ cells were plated 90 µL per well of a 96-well plate on day 1. The treatments were added to each well of the plates on day 2. The viability of cells was measured using 1:1 dilution of the CellTiter-Glo luminescent reagent (Promega G7572), which was read on a Glomax explorer (Promega) after 10 min of shaking at room temperature. The intensity of luminescence was normalized to that of DMSO control. Percentage of Cell death was counted as 100 minus percentage of cell viability.

#### Mouse xenograft

Six weeks old female nude mice (NU/NU, Charles River) were used. All the mice were housed in a temperature controlled room (65–75 °F) with 40–60% humidity, with a light/dark cycle of 12 h/12 h. Mice weights were about 24.7 ± 1.77 g. 2.0 × 10^6^ of A375 or A549 CRISPR control, p53^−/−^, iPLA2β^−/−^, or p53^−/−^; iPLA2β^−/−^ cells were mixed with Matrigel (BD Biosciences) at 1:1 ratio (volume) and injected subcutaneously into nude mice. The mice were killed 20 days for A375, or 30 days for A549 cells after injection and the weight of the tumors was measured and recorded. Every experimental protocol was approved by the Institutional Animal Care and Use Committee (IACUC) of Columbia University (AAAW1456). The tumors in the experiments did not exceed the limit for tumor burden (10% of total body weight or 2 cm in diameter).

### Drugs and inhibitors

For ROS generation, *tert*-Butyl hydroxide solution (TBH, Sigma), butanone peroxide (BTP, Sigma), or cumene hydroperoxide (CMH, Sigma) was used at as seen in respective figure legends. Specific cell-death inhibitors were used in the experiments at the following concentrations: Ferrostatin-1 (ferroptosis inhibitor; Xcess Biosciences), 2 μM; Z-VAD-fmk (Pan-caspase inhibitor; Sigma), 10 μg/mL; Necrostatin-1 (necroptosis inhibitor; Sigma), 10 μg/mL; 3-MA (autophagy inhibitor; Sigma), 2 mM.

### Lipid ROS assay using flow cytometer

Lipid ROS assay was performed as described previously^11^. Briefly, cells were incubated with DMEM containing 5 µM of C-11 BODIPY dye in tissue culture incubator for 30 min. Cells were then collected by trypsinization, and washed twice with PBS followed by re-suspending in 500 µL of PBS. Lipid ROS levels were analyzed using a Becton Dickinson FACSCalibur machine through the FL1 channel. In all, 10,000 cells were analyzed using CellQuest in each sample.

#### LC–MS/MS-based analysis of oxidized phospholipids

H1299 cells were transfected ALOX12 alone or co-transfected ALOX12 plus iPLA2β or iPLA2β enzymatic deficit mutant with three biological replicates. One day after transfection, 1 million cells were trypsinized, washed, and collected in cryogenic vials. Vials were stored at liquid nitrogen until the time of analysis. The cell pellets were thawed on ice and added 500 µL PBS/methanol 1:1 (v/v). Then 100 µL methanol containing 50 ng each of the internal standards PC(15:0/18:1‐d7), PE(15:0/18:1‐d7) (EquiSPLASH, Avanti Polar Lipids). Samples were then transferred into 8 mL screw‐cap tubes, and 1.125 methanol and 5 mL MTBE were added. After vigorous mixing, samples were incubated at room temperature on a tabletop shaker for 45 min. For phase separation, 1.25 mL water was added, and samples were vortexed and centrifuged for 15 min at 2000*g*. The upper organic phase of each sample was carefully removed using a Pasteur pipette, transferred into an empty glass round‐bottom tube, and dried under vacuum in a SpeedVac concentrator. The dried lipid extracts were resuspended in 200 µL HPLC mobile phase A/mobile phase B 3:1 (v/v) for targeted lipidomic analysis of oxidized phospholipids. 20 µL samples were used for HPLC system (ExionLC Integrated System, Sciex)/Mass spectrometer (QTrap 6500+, Sciex) detection. Here is the index for HPLC: Column: Kinetex 2.6 µm HILIC 100 Å 100 × 2.1 mm, Phenomenex; Flow Rate: 200 µL/min. Total ion signals corresponding to all analytes and internal standards in the positive‐ and negative‐ion modes. The specific precursor–product pairs monitored in negative-ion mode and used for quantification: PE(18:0/20:4), 766.5/303.2; PE(18:0/22:4), 794.6/331.2; PE(18:0/20:4-OH), 782.5/319.2; PE(18:0/20:4-OOH), 798.5/335.2; PE(18:0/22:4-OH), 810.6/347.2; PE(18:0/22:4-OOH), 826.6/363.2; PC(18:0/20:4), 868.6/303.2; PC(18:0/22:4), 896.6/331.2; PC(18:0/20:4-OH), 884.6/319.2; PC(18:0/20:4-OOH), 900.7/335.2; PC(18:0/22:4-OH), 912.6/347.2; PC(18:0/22:4-OOH), 928.6/363.2; PC(15:0/18:1-d7) internal standard, 753.6/184.1; PE(15:0/18:1-d7) internal standard, 711.6/570.3. The relative content of oxidized phospholipids was calculated by the ratio of hydroxyl group (–OH) and hydroperoxyl group (–OOH) to total phospholipids.

### Data analysis and statistics

Data quantification was performed with Excel 2016, ImageJ 1.51W, and CellQuest Pro 6.0. Statistical analyses with Excel and GraphPad Prism V6. Images were processed by Adobe Photoshop. Results are shown as mean ± s.d., except in Fig. 6b, f. Difference was determined by using a two-tailed, unpaired Student’s *t*-test with a confidence interval (CI) of 95%. *p* < 0.05 was defined as statistically significant.

### Reporting summary

Further information on research design is available in the Nature Research Reporting Summary linked to this article.

## Supplementary information

Supplementary Information

Reporting Summary

## Data Availability

The association of iPLA2β with overall survival in patients with kidney renal clear cell carcinoma and acute myeloid leukemia were from cBioPortal for Cancer Genomics databases (http://www.cbioportal.org). All other data for the findings of this study are available from the corresponding author on reasonable request. Source data are provided with this paper.

## Source data

Source Data
