# Supplementary material for: iPLA2β-mediated lipid detoxification controls p53-driven ferroptosis independent of GPX4
Source: Nat Commun. 2021 Jun 15;12:3644. doi: 10.1038/s41467-021-23902-6 (PMC8206155; doi:10.1038/s41467-021-23902-6)
Supplement: Supplementary file 1 — Supplementary Information [file 41467_2021_23902_MOESM1_ESM.pdf]

Supplementary Figure 1

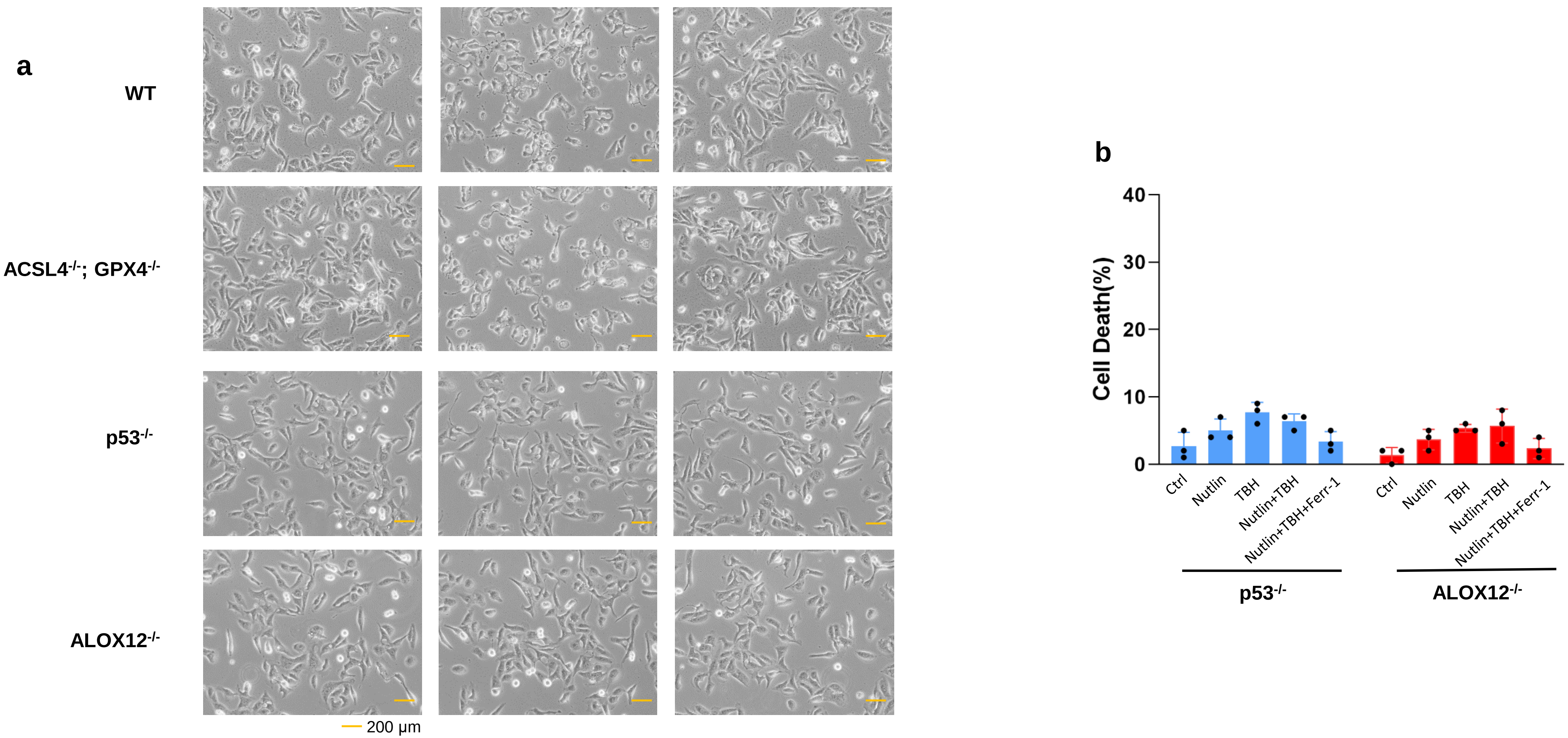

Supplementary Figure 1

a. Representative phase-contrast images of U2OS ctrl, ACSL4$^{-/-}$; GPX4$^{-/-}$, p53$^{-/-}$ and ALOX12$^{-/-}$ cells pre-incubated with Nutlin (10µM) for 24h were treated with TBH (250µM) and Nutlin (10µM) as indicated. The experiments were repeated twice, independently, with similar results.

b. U2OS p53$^{-/-}$ and ALOX12$^{-/-}$ cells pre-incubated with Nutlin (10µM) for 24h were treated with TBH (250µM), Nutlin (10µM) and Ferr-1 (2µM) as indicated. Error bars are mean ± s.d., n=3 independent experiments.

Supplementary Figure 2

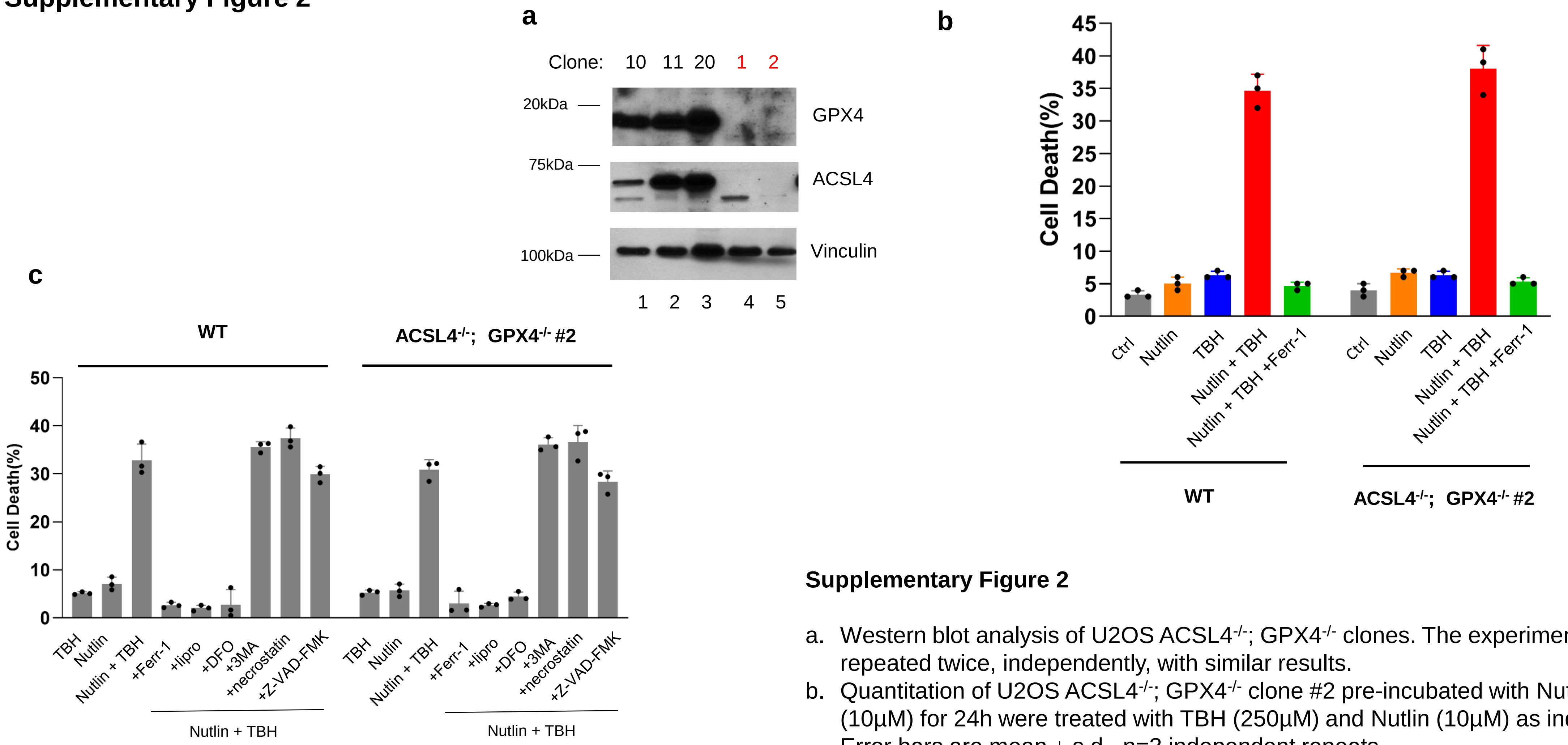

Supplementary Figure 2

a. Western blot analysis of U2OS ACSL4$^{-/-}$; GPX4$^{-/-}$ clones. The experiments were repeated twice, independently, with similar results.

b. Quantitation of U2OS ACSL4$^{-/-}$; GPX4$^{-/-}$ clone #2 pre-incubated with Nutlin (10μM) for 24h were treated with TBH (250μM) and Nutlin (10μM) as indicated. Error bars are mean ± s.d., n=3 independent repeats.

c. U2OS control, ACSL4$^{-/-}$; GPX4$^{-/-}$ cells pre-incubated with Nutlin (10μM) for 24h were treated with TBH (250μM), Nutlin (10μM), Ferr-1 (2μM), Lipro-1 (2μM), 3-MA (2mM), necrostatin-1 (10μg/ml) and Z-VAD-FMK (10μg/ml) as indicated for 8h. Cell death was measured by Cell-Titer-Glo. 3-MA, 3-methylademine. Error bars are mean ± S.D., n= 3 biologically independent experiments.

Supplementary Figure 3

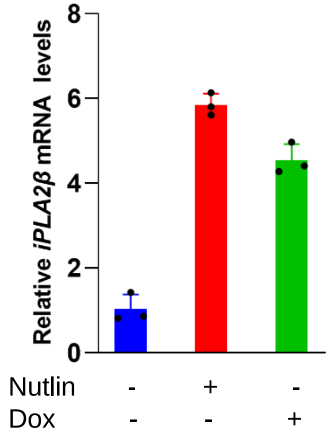

Supplementary Figure 3

qPCR analysis of mRNA levels of *iPLA2β* in the A549 cells treated with 0.2 µg/ml doxorubicin or 10 µM Nutlin for 24 hrs. Error bars are mean ± s.d., n= 3 independent biologically independent experiments.

Supplementary Figure 4

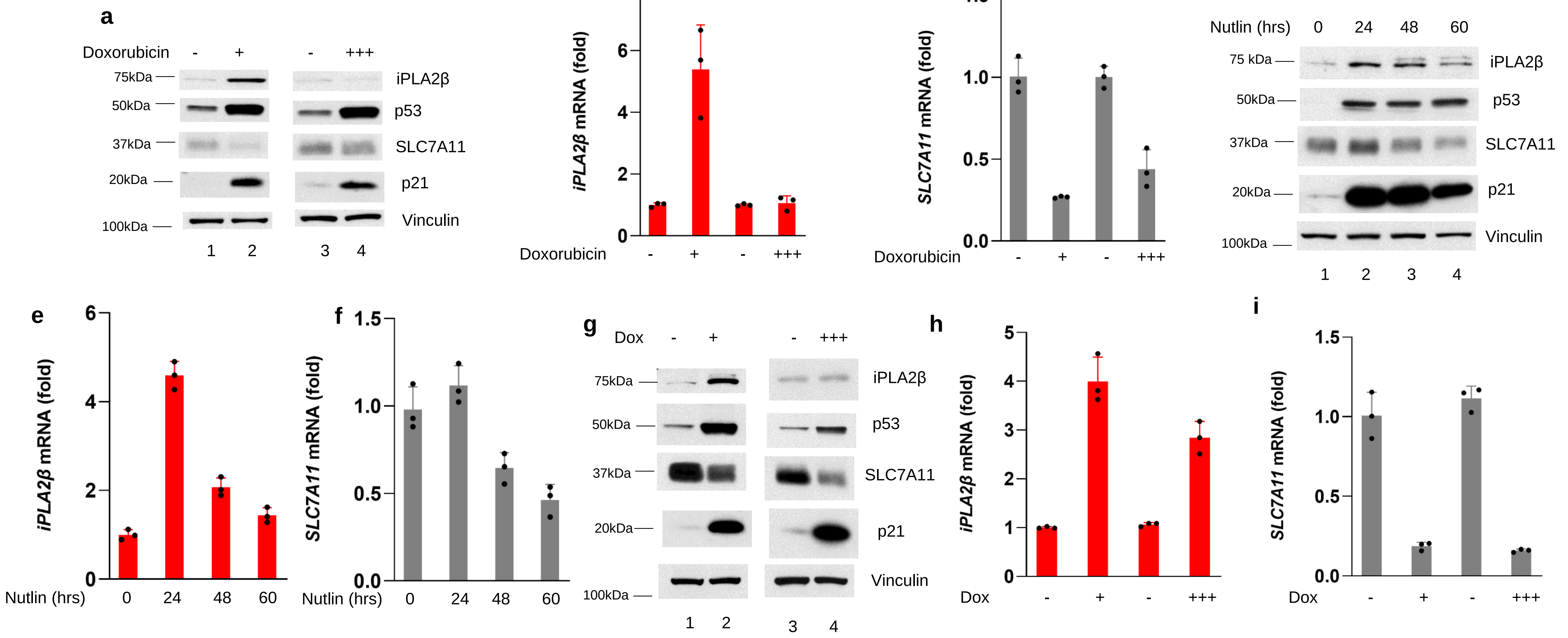

Supplementary Figure 4

a. Western blot analysis of extracts of U2OS cells with low dose (lanes 1, 2) or high dose of doxorubicin (lanes 3, 4) as indicated for 30 hrs. The experiments were repeated twice, independently, with similar results. b,c qPCR analysis of mRNA levels of *iPLA2β* (b) *or SLC7A11* (c) for the U2OS cells in Supplementary Figure 4a. d. Western blot analysis of extracts of A549 cells with different time of 10 µM Nutlin treatment. The experiments were repeated twice, independently, with similar results. e,f qPCR analysis of mRNA levels of *iPLA2β* (e) *or SLC7A11* (f) for the A549 cells in Supplementary Figure 4d. g. Western blot analysis of extracts of A549 cells with low dose (lanes 1, 2) or high dose of doxorubicin (lanes 3, 4) as indicated for 30 hrs. The experiments were repeated twice, independently, with similar results. h,i qPCR analysis of mRNA levels of *iPLA2β* (h) or SLC7A11 (i) for the A549 cells in Supplementary Figure 4g. b-c, e-f, h-I Error bars are mean ± s.d., n= 3 biologically independent experiments.

Supplemental Figure 5

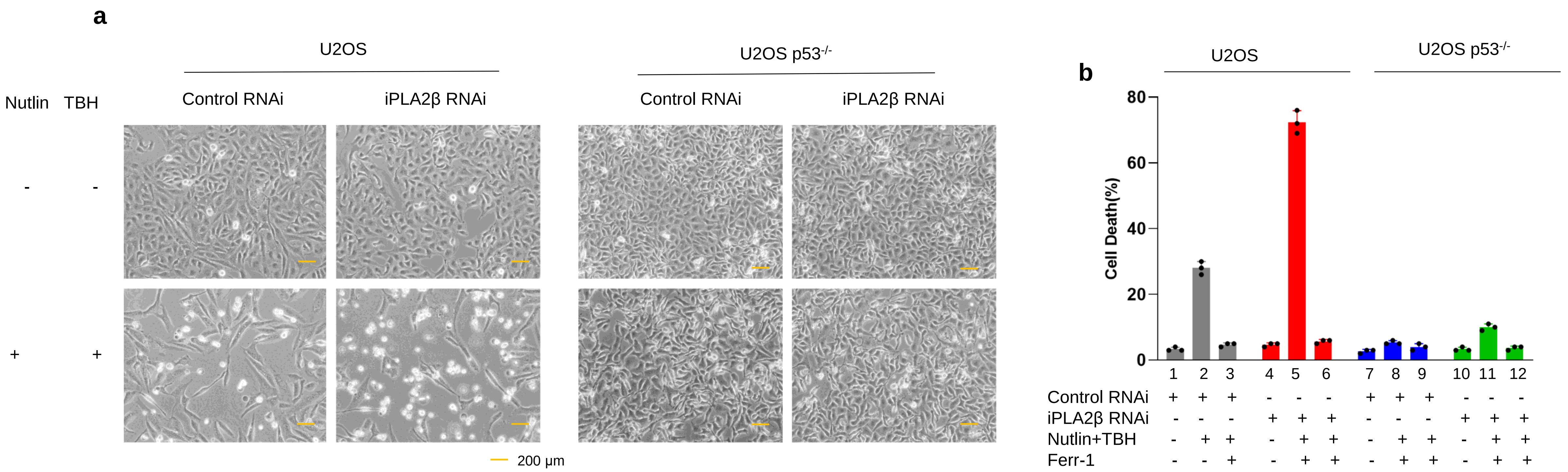

Supplemental Figure 5
a. Representative phase-contrast images of ferroptotic cell death for U2OS CRISPR control versus p53$^{-/-}$ cells treated with control RNAi or iPLA2β RNAi, and with addition of 10 μM Nutlin for 24 hrs by subsequent 250 μM TBH treatment.
b. Quantification of ROS-induced ferroptotic cell death by 250 μM TBH, and 2 μM Ferr-1 treatment as indicated from the same cells as in Supplementary Figure 5a (error bars, s.d. from three independent experiments).

Supplementary Figure 6

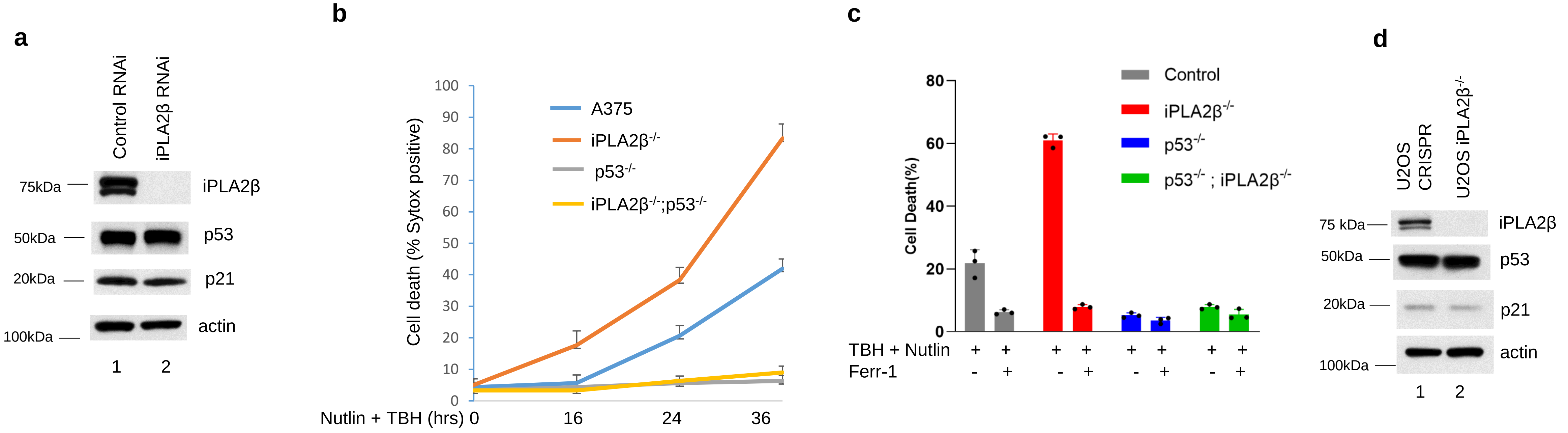

Supplementary Figure 6

a. Western blot analysis of extracts of MCF-7 cells treated with control RNAi (lanes 1) or iPLA2β RNAi (lanes 2) by the antibodies to iPLA2β, p53, p21or actin. The experiments were repeated twice, independently, with similar results.
b. Different time points of cell death for A375 CRISPR cells with Nutlin + TBH treatment. Error bars are mean ± s.d., n= 3 biologically independent experiments.
c. Quantification of cell death by Cell-Titer-Glo in the A375 cells pre-incubated with Nutlin plus additional of 150 µm TBH as indicated. Error bars are mean ± s.d., n= 3 biologically independent experiments.
d. Western blot analysis of extracts of U2OS CRISPR control (lane 1) versus iPLA2β$^{-/-}$ cells (lane 2) by the antibodies to iPLA2β, p53, p21 or actin. The experiments were repeated twice, independently, with similar results.

Supplementary Figure 7

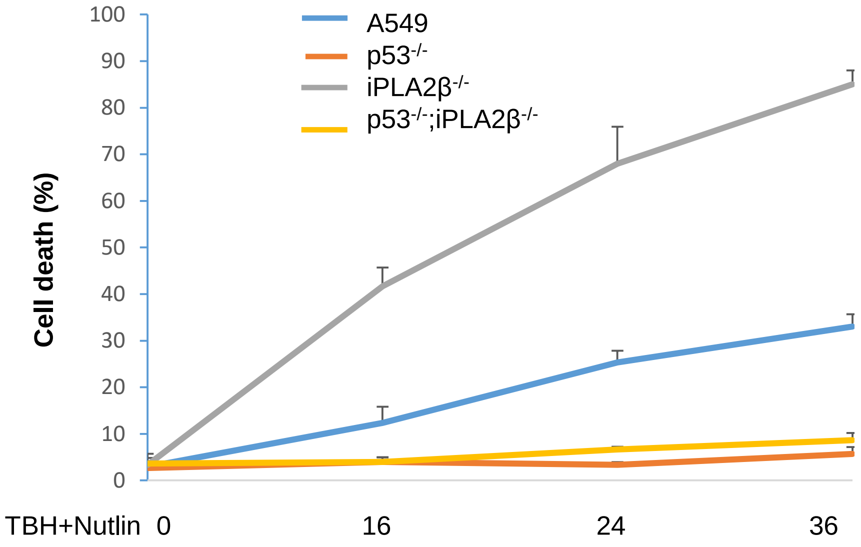

Supplementary Figure 7

Different time points of cell death for A549 CRISPR cells with Nutlin + TBH treatment. Error bars are mean ± s.d., n= 3 biologically independent experiments.

Supplementary Figure 8

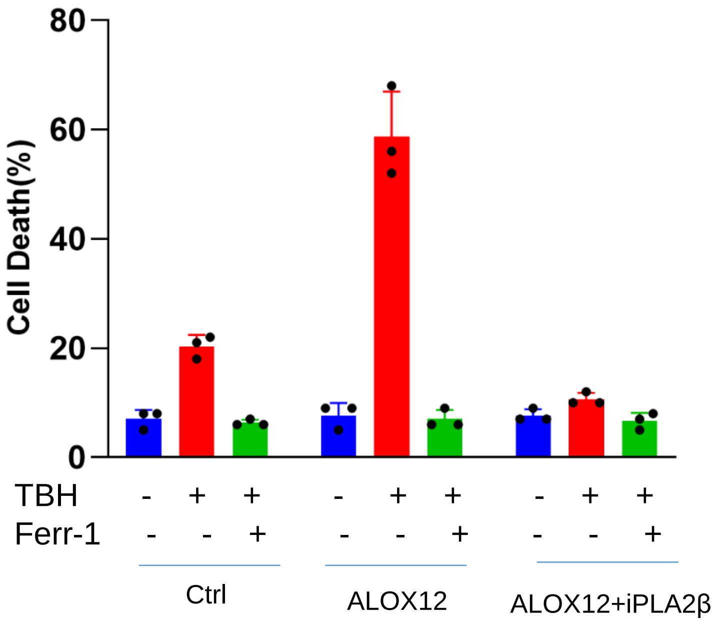

Supplementary Figure 8

Quantification of cell death for the same cells and treatments as described in Figure 7c. Error bars are mean ± s.d., n=3 independent experiments.

Supplementary Figure 9

a

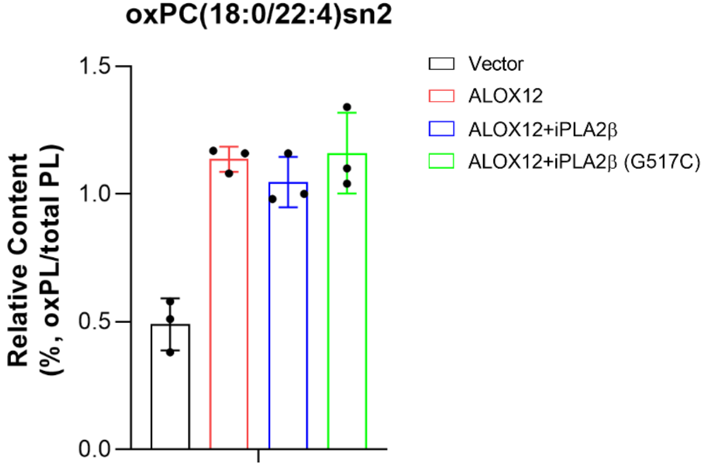

b

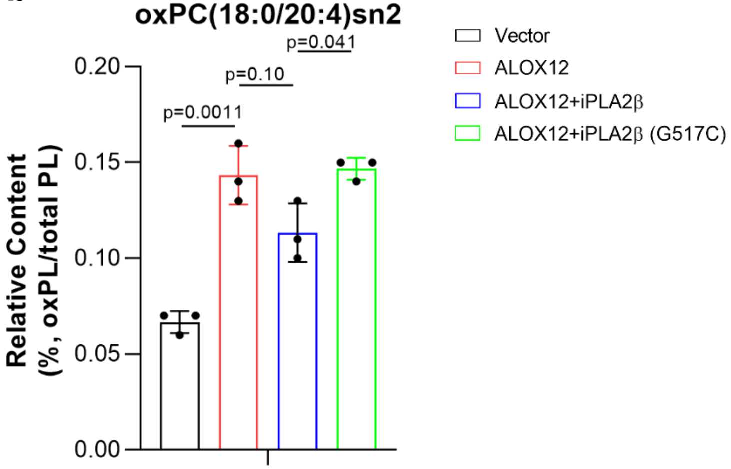

c

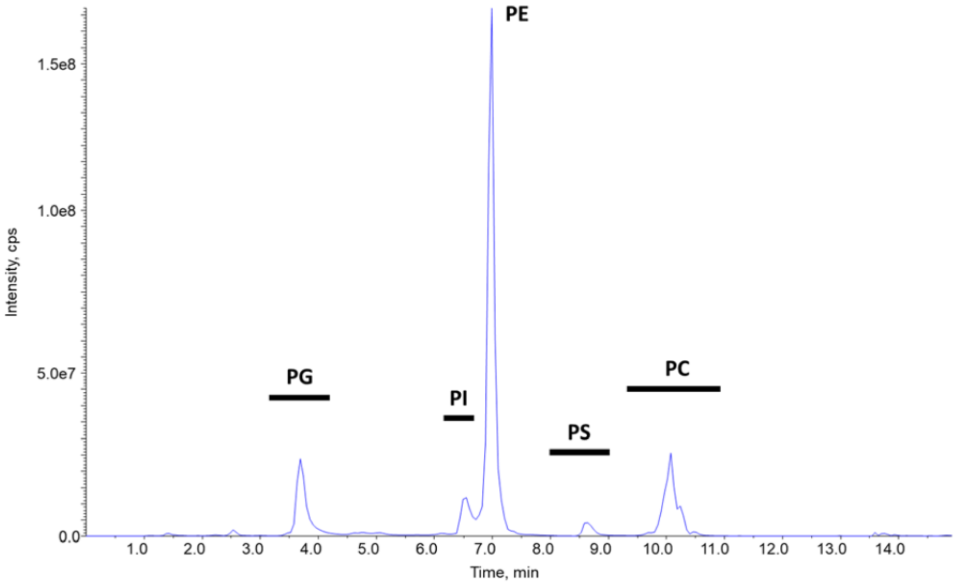

Supplementary Figure 9

a. Relative content of phospholipids oxPC(18:0/22:4)sn2 in H1299 cells transfected with alox12, or/and iPLA2β. (PC, phosphatidylcholine).
b. Relative content of phospholipids oxPC(18:0/20:4)sn2 in H1299 cells transfected with alox12, or/and iPLA2β.
c. Typical LC-MS/MS chromatogram of one representative sample for 5 major classes of phospholipids in H1299 cells. PG, phosphatidylglycerol; PI, phosphatidylinositol; PE, phosphatidylethanolamines; PS, phosphatidylserine; PC, phosphatidylcholine.
a,b Error bars are mean ± s.d., n= 3 biologically independent experiments.

Supplementary Figure 10

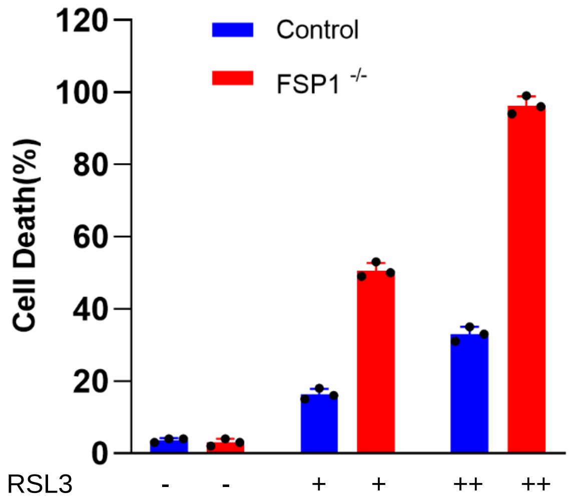

Supplementary Figure 10

Quantification of cell death in the U2OS CRISPR control versus FSP$^{-/-}$ cells treated with RSL3 for 24 hrs. Error bars are mean ± s.d., n= 3 biologically independent experiments.

Supplementary Figure 11

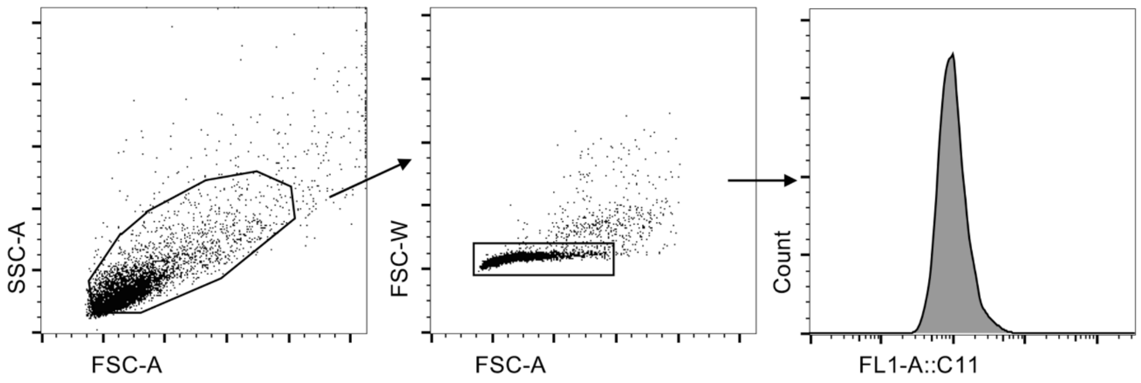

Supplementary Fig. 11

Gating strategy to determine the levels of lipid peroxidation in cells with or without TBH treatment. In the right picture, the gate is used to collect more than 90 percentage of cells; In the middle picture, the gate is used to exclude adhesive cells; The gating panel in the right picture corresponds to FACS data panel presented on Fig. 2d and 7b, and the average signaling intensity of FL1-A::C11 in collected cells corresponds to FACS data presented on Fig. 2e, 7c and 7e.

Supplementary Table 1

Name	Sequence
Human iPLA2β Forward	5’-GCAATGCTCGGTGCAACAT-3’
Human iPLA2β Reverse	5’-ACACCCCTTCTGAGAGAACTTCA-3’
Human HPRT Forward	5'-TATGGCGACCCGCAGCCCT-3'
Human HPRT Reverse	5'-CATCTCGAGCAAGACGTTCAG-3'
iPLA2β primers	
RE1 Forward	5’-GCCGGCTCTGTATCTCTCAA-3’
RE1 Reverse	5’-ACCGAATGACCCTGGGAAGG-3’
RE2 Forward	5’-CCTCTGCCTTCTGGGCTTAA-3’
RE2 Reverse	5’-ACATGGTGAAACCCCATCTC-3’
RE3 Forward	5’-GCATCACTGGTCTCTGTCGC-3’
RE3 Reverse	5’-GTCTAAAATGGGGTTCTGCT-3’
TIGAR primers	
Forward	5′-CGGCAGGTCTTAGATAGCTT-3’
Reverse	5′-GGCAGCCGGCATCAAAAACA-3’
