# Supplementary material for: iPLA2β-mediated lipid detoxification controls p53-driven ferroptosis independent of GPX4
Source: Nat Commun. 2021 Jun 15;12:3644. doi: 10.1038/s41467-021-23902-6 (PMC8206155; doi:10.1038/s41467-021-23902-6)
Supplement: Supplementary file 2 — Reporting Summary [file 41467_2021_23902_MOESM2_ESM.pdf]

nature research

Corresponding author(s): Wei Gu

Last updated by author(s): 05/20/2021

Reporting Summary

Nature Research wishes to improve the reproducibility of the work that we publish. This form provides structure for consistency and transparency in reporting. For further information on Nature Research policies, see Authors & Referees and the Editorial Policy Checklist.

Statistics

For all statistical analyses, confirm that the following items are present in the figure legend, table legend, main text, or Methods section.

n/a	Confirmed	
☐	☒	The exact sample size (*n*) for each experimental group/condition, given as a discrete number and unit of measurement
☐	☒	A statement on whether measurements were taken from distinct samples or whether the same sample was measured repeatedly
☐	☒	The statistical test(s) used AND whether they are one- or two-sided<br>*Only common tests should be described solely by name; describe more complex techniques in the Methods section.*
☒	☐	A description of all covariates tested
☒	☐	A description of any assumptions or corrections, such as tests of normality and adjustment for multiple comparisons
☐	☒	A full description of the statistical parameters including central tendency (e.g. means) or other basic estimates (e.g. regression coefficient) AND variation (e.g. standard deviation) or associated estimates of uncertainty (e.g. confidence intervals)
☐	☒	For null hypothesis testing, the test statistic (e.g. *F*, *t*, *r*) with confidence intervals, effect sizes, degrees of freedom and *P* value noted<br>*Give P values as exact values whenever suitable.*
☒	☐	For Bayesian analysis, information on the choice of priors and Markov chain Monte Carlo settings
☒	☐	For hierarchical and complex designs, identification of the appropriate level for tests and full reporting of outcomes
☒	☐	Estimates of effect sizes (e.g. Cohen's *d*, Pearson's *r*), indicating how they were calculated

Our web collection on statistics for biologists contains articles on many of the points above.

Software and code

Policy information about availability of computer code

Data collection	Becton Dickinson FACSCalibur machine was used to acquire and analyze flow cytometry data. Cell death was recorded with the Countess II (Life Technologies). For CellTiter-Glo luminescent assay, a Glomax explorer (Promega) was used.
Data analysis	Data quantification were performed with Excel 2016, Image J 1.51W and CellQuest Pro. 6.0, and statistical analyses with Excel and GraphPad Prism V6. Images were processed by Adobe Photoshop.

For manuscripts utilizing custom algorithms or software that are central to the research but not yet described in published literature, software must be made available to editors/reviewers. We strongly encourage code deposition in a community repository (e.g. GitHub). See the Nature Research guidelines for submitting code & software for further information.

Data

Policy information about availability of data

All manuscripts must include a data availability statement. This statement should provide the following information, where applicable:

- Accession codes, unique identifiers, or web links for publicly available datasets
- A list of figures that have associated raw data
- A description of any restrictions on data availability

The association of iPLA2β with overall survival in patients with Kidney renal Clear Cell Carcinoma and Acute Myeloid Leukemia were from Bioportall for Cancer Genomies databases (http://www.cbioportal.org). The associated raw data and statistic analysis as applicable for Figs. 1-8, and Supplementary Figs 1-10 are provided as Source Data Files. All other data for the findings of this study are available from the corresponding author on reasonable request.

Field-specific reporting

Please select the one below that is the best fit for your research. If you are not sure, read the appropriate sections before making your selection.

☒ Life sciences ☐ Behavioural & social sciences ☐ Ecological, evolutionary & environmental sciences

For a reference copy of the document with all sections, see nature.com/documents/nr-reporting-summary-flat.pdf

Life sciences study design

All studies must disclose on these points even when the disclosure is negative.

Sample size	The sample size was determined based on the experiments. No sample size calculation was performed for this study. Based on the literature reported, 3 independent samples were chosen for the experiments, and 8 xenografts were chosen for the animal study to achieve Student's t-test analysis.
Data exclusions	No data was excluded from this study.
Replication	Figs 1a, 4a, 4d, 4g, 5a, 5d, 6a, 6d, 6e, 7a, 7d, 8a, Supplementary Figs 2a, 4a, 4d, 4g, 6a, 6d were repeated twice. All the other experiments were repeated independently three times.
Randomization	6 week female nude mice were chosen for xenograft models, and randomly allocated into experimental groups. For experiments other than animal studies, samples were allocated randomly into experimental groups too.
Blinding	For cell-based in vitro experiments, the investigators were not blinded on data acquisition and analysis. The application of treatments and processing procedures were difficult for blinding but there was no human bias given all the data were collected independently using instrumentation. For the animal study the investigators were not blinded to the experimental group allocation. We only did animal study for xenograft. The final persons for measure tumors are blinded to the experimental group allocation. Also at least two observers measured xenograft tumor weights to alleviate human bias in this study. Moreover, measure of weight was very objective, and bias is minimal.

Reporting for specific materials, systems and methods

We require information from authors about some types of materials, experimental systems and methods used in many studies. Here, indicate whether each material, system or method listed is relevant to your study. If you are not sure if a list item applies to your research, read the appropriate section before selecting a response.

Materials & experimental systems

n/a	Involved in the study	
☐	☒	Antibodies
☐	☒	Eukaryotic cell lines
☒	☐	Palaeontology
☐	☒	Animals and other organisms
☒	☐	Human research participants
☒	☐	Clinical data

Methods

n/a	Involved in the study	
☒	☐	ChIP-seq
☐	☒	Flow cytometry
☒	☐	MRI-based neuroimaging

Antibodies

Antibodies used	p53 (sc126, DO-1; Santa Cruz, 1:1000 dilution), iPLA2β (sc376563, D-4, Santa Cruz, 1: 200 dilution), MDM2 (SRP2095, Ab5; Millipore, 1: 500 dilution), p21 (C-19; Santa Cruz, 1: 400 dilution), ACSL4 (sc-271800; Santa Cruz, 1: 1000 dilution), GPX4 (ab125066; Abcam, 1:500 dilution), V5 (46-0725; Invitrogen, 1: 500 dilution), ALOX12 (sc-365194; Santa Cruz, 1:200 dilution), AMID (FSP1) (sc-377120; Santa Cruz, 1:400 dilution), SLC7A11 (D2M7A) (12691s; Cell Signaling, 1: 500 dilution), Vinculin (V9264; Sigma-Aldrich, 1: 5000 dilution), Actin (A3853; Sigma-aldrich, 1 : 5000 dilution), GFP (565271; BD Biosciences, 1: 1000 dilution) and TIGAR (sc-166290, Santa Cruz, 1:500 dilution), HRP-conjugated anti-mouse (111-035-146, Jacksonimmuno, 1:500 dilution), HRP-conjugated anti-rabbit (111-035-045, Jacksonimmuno, 1:500 dilution).
Validation	All antibodies used were validated by their commercial source for the application used.<br>p53(DO-1), https://www.scbt.com/scbt/product/p53-antibody-do-1;<br>iPLA2β, https://www.scbt.com/p/group-vi-ipla2-antibody-d-4<br>MDM2, http://www.emdmillipore.com/US/en/product/Anti-MDM2-Ab-5-Mouse-mAb-4B2C1.11,EMD_BIO-OP145;<br>p21, https://www.scbt.com/scbt/product/p21-antibody-sx118;<br>ACSL4, https://www.scbt.com/scbt/product/acsl4-antibody-a-5;<br>GPX4, https://www.abcam.com/glutathione-peroxidase-4-antibody-epncir144-ab125066.html;<br>V5, https://www.thermofisher.com/antibody/product/V5-Tag-Antibody-Monoclonal/R960-25;

AMID, https://www.scbt.com/p/amid-antibody-b-6?requestFrom=search;
SLC7A11, https://www.cellsignal.com/products/primary-antibodies/xct-slc7a11-d2m7a-rabbit-mab/12691;
Vinculin, https://www.sigmaaldrich.com/catalog/search?term=v9264&interface=All&N=0&mode=match%20partialmax&lang=en&region=US&focus=product
Actin, https://www.sigmaaldrich.com/catalog/search?term=a3853&interface=All&N=0&mode=match%20partialmax&lang=en&region=US&focus=product
TIGAR, https://www.scbt.com/p/tigar-antibody-e-2
GFP, https://www.bdbiosciences.com/eu/reagents/research/antibodies-buffers/cell-biology-reagents/cell-biology-antibodies/biotin-mouse-anti-gfp-1a12-6-18/p/565271
HRP-conjugated anti-mouse, https://www.jacksonimmuno.com/catalog/products/115-035-146
HRP-conjugated anti-rabbit, https://www.jacksonimmuno.com/catalog/products/111-035-045

Eukaryotic cell lines

Policy information about cell lines

Cell line source(s)	U2OS, A549, A375, HCT116, H1299 and MCF-7 cancer lines were purchased from American Type Culture Collection (ATCC) and have been proven to be negative for mycoplasma contamination.
Authentication	All cell lines were not authenticated.
Mycoplasma contamination	The cell lines were tested negative for mycoplasma contamination
Commonly misidentified lines (See ICLAC register)	No cell line used in this study was found in the database of commonly misidentified cell lines that are maintained by ICLAC and NCBI biosample

Animals and other organisms

Policy information about studies involving animals; ARRIVE guidelines recommended for reporting animal research

Laboratory animals	NU/J nude female mice at 6 week old purchased from Charles River were used for Xenograft model. All the mice were housed in a temperature controlled room (65- 75 °f) with 40–60% humidity, with a light/dark cycle of 12 h/12 h.
Wild animals	The study did not use any wild animal
Field-collected samples	No field-collected sample was used in the study
Ethics oversight	The study is compliant with all the relevant ethical regulations for animal experiments. All experimental protocols were approved by the Institutional Animal Care and Use Committee of Columbia University

Note that full information on the approval of the study protocol must also be provided in the manuscript.

Flow Cytometry

Plots

Confirm that:

☒ The axis labels state the marker and fluorochrome used (e.g. CD4-FITC).

☒ The axis scales are clearly visible. Include numbers along axes only for bottom left plot of group (a 'group' is an analysis of identical markers).

☒ All plots are contour plots with outliers or pseudocolor plots.

☒ A numerical value for number of cells or percentage (with statistics) is provided.

Methodology

Sample preparation	The cells were incubated with medium containing 5 µm of BODIPY-C11 dye for 20 min. Then the cells were harvested and washed twice with PBS followed by re-suspending in 500 µl of PBS
Instrument	Becton Dickinson FACSCalibur machine
Software	CellQuest
Cell population abundance	10,000 cells were analyzed for each sample
Gating strategy	The initial cell population was gated by Forward Scatter and Side Scatter to make sure double exclusion. Only single cell was used for analysis. Details of gating strategy to determine the levels of lipid peroxidation in cells were shown in Supplementary Figure 11

☒ Tick this box to confirm that a figure exemplifying the gating strategy is provided in the Supplementary Information.
